# Validation of semiquantitative FFQ administered to adults: a systematic review

**DOI:** 10.1017/S1368980020001834

**Published:** 2021-08

**Authors:** Érika Sierra-Ruelas, María F Bernal-Orozco, Gabriela Macedo-Ojeda, Yolanda F Márquez-Sandoval, Martha B Altamirano-Martínez, Barbara Vizmanos

**Affiliations:** 1 Doctorado en Ciencias de la Nutrición Traslacional, Centro Universitario de Ciencias de la Salud (CUCS), Universidad de Guadalajara (UdeG), Juan Díaz Covarrubias y Salvador Quevedo y Zubieta, ZC 44340 Guadalajara, Jalisco, Mexico; 2 Licenciatura en Nutrición, CUCS, UdeG, Sierra Mojada 950, ZC 44340 Guadalajara, Jalisco, México

**Keywords:** semiquantitative FFQ, Validity, Validation, Reproducibility, Systematic review

## Abstract

**Objective:**

To conduct a systematic review of studies for the validation of semiquantitative FFQ (SFFQ) that assess food intake in adults.

**Design:**

The authors conducted a systematic search in PubMed for articles published as late as January 2020 in Spanish, English, French and Portuguese. Individual searches (twelve in total) paired three hyphenated and non-hyphenated variations of ‘semiquantitative food frequency questionnaire’ with both ‘validity’ and ‘validation’ using the ‘all fields’ and the ‘title/abstract’ retrieval categories. Independent extraction of articles was performed by four authors using predefined data fields.

**Setting:**

We searched for original SFFQ validation studies that analysed general diet composition (nutrients with or without food groups or energy analysis) in healthy adults, in any setting, and that also reported correlation coefficients.

**Participants:**

Healthy adults.

**Results:**

Sixty articles were included. The preferred comparison standard for validation was food records (*n* 37). The main correlation coefficients used were Pearson’s (*n* 41), and validity coefficients varied from −0·45 to 1. Most correlation coefficients were adjusted by energy (twelve studies presented only crude values). The elements mentioned most frequently were energy, macronutrients, cholesterol, SFA, PUFA, fibre, vitamin C, Ca and Fe.

**Conclusions:**

Although all these SFFQ are reported as validated, coefficients may vary across groups of foods and nutrients. Based on our findings, we suggest researchers to consult our revision before choosing a SFFQ and to review important issues about them, such as their validation, number of items, number of participants, etc. Systematic Review Registration: PROSPERO number CRD42017064716. Available at: http://www.crd.york.ac.uk/PROSPERO/display_record.asp?ID=CRD42017064716.

Given the influence that food can have on the development, prevention and treatment of diseases, having a thorough knowledge of a population’s food habits is of vital importance. However, measurements of dietary intake are difficult to perform and are thus considered as one of the major methodological challenges within the field of nutritional epidemiology^(1)^.

Currently, semiquantitative FFQ (SFFQ) are considered to be an important method of obtaining data on the long-term habitual intake patterns of large populations^(2)^. The main objective of SFFQ is to assess diet over long periods of time. They have been used for research on epidemiology and non-communicable diseases, as well as in studies focused on specific foods or nutrients^(3)^. In addition, the SFFQ is considered to be a relatively inexpensive, quick and easy-to-implement method that can provide in-depth insights into food and nutrient intake and dietary patterns^(1,4)^.

Unlike qualitative FFQ, SFFQ include specific portion sizes in their questions or items on food intake frequency^(3)^ and they also require weighted responses regarding these portions. Their overall aim is to obtain estimates of nutrient intake, which may help to identify dietary deficiencies or excesses.

However, because a SFFQ that has been developed in a particular society and culture may not be applicable elsewhere, first it must be validated in the population for which it has been designed^(1,5)^, given that validity is a continuous variable which may range from no validity to very high^(3)^. Besides, because SFFQ responses are based on memories of previous eating habits, administering them to children and the elderly may be particularly problematic. Hence, they are generally and should preferably be administered to healthy adults when validating the instrument for use on the general population^(5)^.

Because of the considerable variety of available SFFQ, it may be challenging to select the best one for a context. Thus, this systematic review of SFFQ validation studies to assess food intake in adults is intended to serve as an up-to-date reference tool that will help researchers to validate SFFQ or choose those best suited to the needs of whatever specific studies they wish to conduct on particular populations.

## Methods

This study is a systematic review that followed criteria for the search and selection of articles stipulated by the Preferred Reporting Items for Systematic Reviews and Meta-Analyses (PRISMA) statement^(6)^. Also, the protocol for this study was registered in the International Prospective Register of Systematic Reviews (PROSPERO) with registration number CRD42017064716.

### Literature search

Two authors (E.S.-R. and M.F.B.-O.) performed a systematic search in the PubMed bibliographic index for articles in four languages. Individual searches (twelve in total) paired all possible combinations of the multi-word terms ‘semiquantitative food frequency questionnaire’, ‘semi-quantitative food frequency questionnaire’ and ‘semiquantitative food-frequency questionnaire’ with ‘validity’ and ‘validation’. Each of these searches was performed using both the ‘all fields’ and the ‘title/abstract’ retrieval categories. Results included articles published up to the date of the last search (31 January 2020).

### Study selection

Articles were chosen based on six inclusion criteria: (1) be original SFFQ validation articles; (2) analyse nutrient intake, with or without energy or food groups analysis, and without an exclusive focus on specific nutrients (e.g. folic acid or Fe); (3) include healthy adults with no particular nutritional needs (pregnant women, athletes, etc.); (4) include individuals without cognitive impairment (when elderly subjects are studied); (5) include analyses with correlation coefficients (Spearman’s, Pearson’s and intraclass); (6) be published in English, Spanish, French or Portuguese. Exclusion criteria were studies that (1) validate SFFQ designed for specific diseases (e.g. cancer) or conditions (e.g. post-myocardial infarction); (2) report only food group analyses; (3) describe data only in tertiles, quartiles or quintiles without reporting correlation coefficients and (4) include data reported in a previous paper.

Abstracts obtained using these search criteria were assessed for eligibility according to inclusion criteria and retrieved through the University of Guadalajara’s virtual library and databases, open access links or Google Scholar. If a full-text paper could not be retrieved through these means, it was obtained by contacting the paper’s corresponding author or through the payment of applicable fees. Related articles identified when searching full-text papers were also retrieved, after confirming their adherence to the inclusion criteria and their presence in PubMed. The full text of these studies was also assessed for eligibility. Any doubts about the eligibility of studies were resolved through discussions with a third author (B.V.). Table 1 shows criteria for participants, outcomes and study design (PICOS statement: population [P], outcomes [O] and study design [S]; intervention [I] and comparator [C] are non-applicable since we did not search for clinical trial data).

**Table 1:** PICOS (participants, intervention, comparator, outcome, study design) criteria for study selection

Parameter	Criteria
Population	Inclusion criteria: Studies must: (1) be original ones on SFFQ validation processes; (2) analyse nutrient and energy intake, with or without food group analysis, and without an exclusive focus on specific nutrients; (3) include healthy adults as subjects with no special nutritional needs; (4) have study populations composed of elderly individuals without cognitive impairment (in studies conducted on this population subgroup); (5) include analyses with correlation coefficients and (6) be published in either English, Spanish, French or Portuguese. Exclusion criteria: Studies must not: (1) validate SFFQ designed for specific diseases (e.g. cancer) or conditions (e.g. post myocardial infarction); (2) report only food group analyses; (3) present data only in tertiles, quartiles or quintiles without reporting correlation coefficients and (4) include data reported in a previous paper.
Intervention or exposure	Non-applicable
Comparator	Non-applicable
Outcome	Primary outcomes: reference method used for validation of SFFQ, correlation coefficients for energy, nutrients and/or food groups; inclusion of reproducibility analysis, correlation coefficients reported for reproducibility, time frame for reproducibility assessment. SFFQ characteristics (number of items, number of intake frequency response categories, inclusion of visual support, administration method). Secondary outcomes: participant characteristics (sex of participants, age ranges or mean and standard deviation), validation studies characteristics (year of publication, country, region or continent, number of participants).
Study design	Validation studies

SFFQ, semiquantitative FFQ.

### Data extraction

Independent data extraction from articles was performed by four authors (E.S.-R., M.F.B.-O., G.M.-O. and M.B.A.-M.) in a non-blinded way, into an Excel form for evidence synthesis.

For each SFFQ, the following characteristics were analysed: author and year of publication, country in which the questionnaire was developed, geographical region, number and sex of participants, minimum and maximum ages or age range (difference between the extremes), number of items, number of response categories, visual support to identify portion sizes and how SFFQ were administered (by interview or self-administration). For the analysis of data used in the validation, the following was recorded: validation methods (records and recalls) and the number of times they used; units of analysis (energy, nutrients and food groups) with the lowest and highest correlation coefficient values that were produced (Pearson’s, Spearman’s, intraclass and Rosner’s); and the specific values for energy, carbohydrates, proteins, fat and nutrients that were mentioned most frequently across studies (Ca, Fe, etc.). To determine which nutrients were most frequently reported, a matrix was created in Excel into which the nutrients reported in the studies were captured and where the seven most mentioned nutrients were highlighted. Regarding reproducibility, the intervals between questionnaire administrations and correlation coefficients (Pearson’s, Spearman’s, intraclass and Rosner’s) were identified.

### Quality assessment

Risk of bias analysis was conducted applying elements of three tools: Newcastle–Ottawa scale for cohort studies^(7)^, the Cochrane evaluation tool^(8)^ and some aspects considered in the Strobe statement^(9)^. The Newcastle–Ottawa scale was used to assess: the representativeness of the sample (not volunteers); the assurance of exposure (data obtained from the instruments used to validate the SFFQ were mainly through reminders or records, not self-reporting); whether the analyses were controlled by at least one additional variable (e.g. energy); the method used to assess the results of the SFFQ (preferably by interview); the duration of the follow-up to validate the tool (ideally 6–12 months) and whether it was representative of the habitual diet; and the number of dropouts during the follow-up phase (ideally <30 % of the population) with explanations of the reasons. Cochrane’s tool was used to assess the risk of notification bias (considered as low when the results were consistent with those described in the methodology section). Finally, based on the Strobe Statement, some aspects of the methodology (description of the location, dates, recruitment periods, and eligibility criteria; a detailed description of the SFFQ application method; the way in which the sample size was determined; a description of how variables were addressed in the analysis) and the results (description of participant characteristics and confounding variables and reports of other analyses such as de-attenuation) were evaluated. A total of fourteen factors related to the risk of bias were assessed in each of the studies.

## Results

### Study selection

**Fig. 1:**
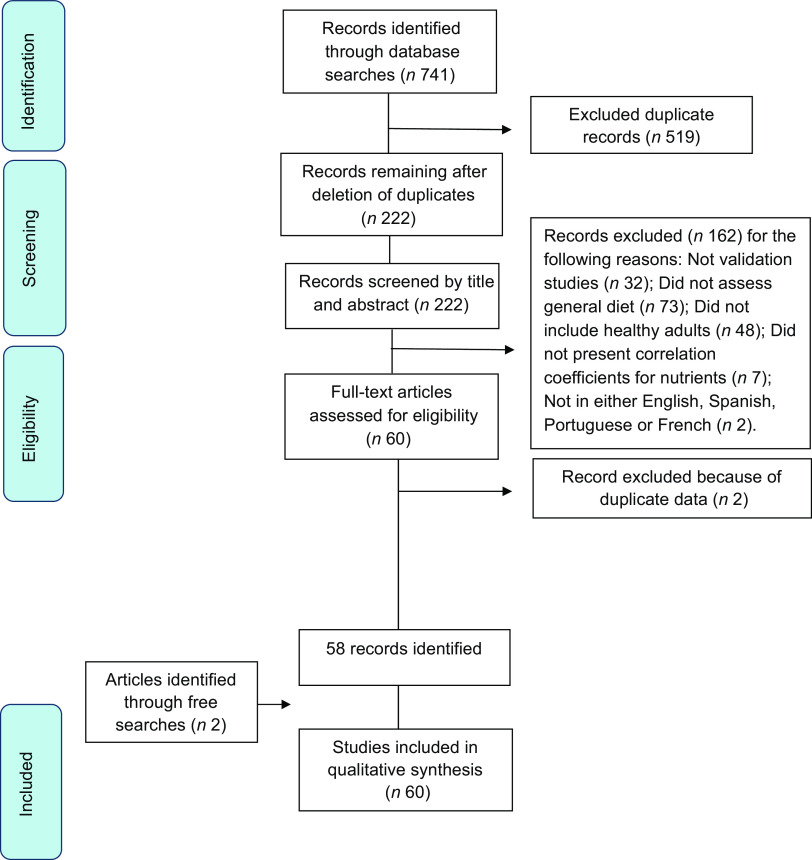
Flow chart of the selection process for studies

Using the above-described search strategy, we initially found 741 articles. After deleting duplicates, this number was reduced to 222. Of these, a total of sixty articles were selected from the bibliographic search, excluding two^(10,11)^ which reported data described in previous papers^(12,13)^ (duplicate data). We added another two articles, which appeared as related articles during full-text searches. We included these articles, which were retrieved from the Internet because they had been published in journals cited in PubMed and met our inclusion criteria. A total of sixty articles were thus included in the analysis. The flow chart for the selection of the articles is shown in Fig. 1.

### Studies’ and participants’ characteristics

Table 2 shows the characteristics of the included validation studies. The selected studies had been published in six geographical categories: twenty-five in Europe^(5,12,14–36)^, seventeen in the Americas^(13,37–52)^, eleven in Asia^(53–63)^, five in Oceania^(64–68)^, one in Africa^(69)^ and one article had origins in both Asia and Europe^(70)^. The publication periods of the articles found were as follows: fifteen were published from 1985 to 1995^(5,12,13,21,22,25,27,33,36,39,42,46,50,51,66)^; nineteen were published from 1996 to 2005^(14,16,17,20,23,26,30,34,35,38,40,41,47–49,54,57,60,67)^; and twenty-six were published from 2006 to January 2020^(15,18,19,24,28,29,31,32,37,43–45,52,53,55,56,58,59,61–65,68–70)^.

**Table 2.** Characteristics of the population and of the SFFQs validated in the studies included in the review, ordered chronologically.

Reference	Year of publication	Country	*n*	Sex	Age range or mean age (SD) or median [IQR]	Number of items	Number of intake frequency response categories	Survey administration method
Willet *et al.* ^(42)^	1985	USA	173	W	34–59	61	9	Interview
Willet *et al.* ^(13)^	1987	USA	27	B	20–54	116	9	Self-administered*†
Tjønneland *et al.* ^(27)^	1991	Denmark	144	B	40–64	92	ND	Self-administered*‡
Rimm *et al.* ^(39)^	1992	USA	127	M	40–75	131	9	Self-administered
Horwath^(66)^	1993	New Zealand	53	B	54–86	120	5 and open format§	Self-administered*‖
Longnecker *et al.* ^(51)^	1993	USA	138	B	22–82	116	9	Self-administered
Martín *et al.* ^(5)^	1993	Spain	147	W	18–74	118	ND	Self-administered
Feskanich *et al.* ^(46)^	1994	USA	127	M	40–75	45	4	Self-administered
Lee *et al.* ^(50)^	1994	USA	74	W	30–60	84	9	Interview*¶
Porrini *et al.* ^(21)^	1994	Italy	30	B	28.5 (6.5)	ND	ND	Self-administered
Ramon *et al.* ^(22)^	1994	Spain	58	B	13–70	39	Open format§	Interview
Rothenberg^(25)^	1994	Italy	46	B	19–47	93	ND	Interview*‖
Fidanza *et al.* ^(33)^	1995	Italy	46	B	19–47	93	Daily, weekly and/or monthly, with the stated number of times (1–6)	Self-administered*‖
Gnardellis *et al.* ^(12)^	1995	Greece	80	B	25–67	190	Open format§	Self-administered*‖
Grootenhuis *et al.* ^(36)^	1995	Netherlands	74	B	50–75	75	11 and open format§	Self-administered
Bonifacj *et al.* ^(30)^	1997	France	98	B	41.9 (11.8)	134	6	Self-administered
Friis *et al.* ^(35)^	1997	Denmark	122	W	20–29	ND	9	Self-administered
Kumanyika *et al.* ^(49)^	1997	USA	96	B	66–100	99	5	Interview*‖
Ocké *et al.* ^(20)^	1997	Netherlands	121	B	20–70	178	Open format§	Self- administered*‖
Hérnandez *et al.* ^(47)^	1998	Mexico	134	W	ND	116	9	Self-administered
Klipstein *et al.* ^(14)^	1998	Netherlands	80	B	55–75	170	Open format§	Both
Smith *et al.* ^(67)^	1998	Australia	79	B	63–80	145	9	Self-administered
Fregapane and Asensio^(34)^	2000	Spain	38	B	18–61	202	Open format§	Self-administered
Jackson *et al.* ^(48)^	2001	Jamaica	123	B	25–74	70	8	Interview*¶
Schröder *et al.* ^(26)^	2001	Spain	44	B	30.7 (10.4)	157	ND	Self-administered
Tokudome *et al.* ^(60)^	2001	Japan	79	W	32–66	102	8	Self-administered**
Rodríguez *et al.* ^(40)^	2002	Guatemala	73	B	22–55	52	Open format§	Interview
Masson *et al.* ^(16)^	2003	United Kingdom	81	B	19–58	150	Open format§	Self-administered*‖
Moreira *et al.* ^(17)^	2003	Portugal	246	B	18–29	89	9	Interview*‖
Chen *et al.* ^(54)^	2004	Bangladesh	189	B	17–75	39	Open format§	Interview*†
Ke *et al.* ^(57)^	2005	China	100	B	M: 41.8 (6); W: 40.9 (5.9)	125	7	ND
Nath and Huffman^(38)^	2005	USA	20	B	53.3 (17.1)	131	9	ND
Roddam *et al.* ^(23)^	2005	United Kingdom	202	W	50–64	87	Multiple choice answers	Self-administered
Shatenstein *et al.* ^(41)^	2005	Canada	248	ND	18–82	74	ND	Self-administered*‖
Dumartheray *et al.* ^(31)^	2006	Switzerland	44	W	75–87	110	9	Self-administered
Sudha *et al.* ^(59)^	2006	India	102	B	40.9 (12.8)	222	Open format§	Interview*†
Nöthlings *et al.* ^(19)^	2007	Germany	393	B	M: 59.0 (8.0); W: 55.0 (10.0)	102	ND	Self-administered
Mullie *et al.* ^(18)^	2009	Belgium	95	M	43.7 (6.6)	150	9	ND
Barret and Gibson^(64)^	2010	Germany	72	B	23–72	297	ND	Self-administered
Fernández *et al.* ^(32)^	2010	Spain	158	B	55–80	137	9	Self-administered
Yang *et al.* ^(61)^	2010	Korea	124	B	40–70	103	9	Interview*‖
Fayet *et al.* ^(65)^	2011	Australia	256	F	18–35	235	9	Self-administered**
van Dongen *et al.* ^(28)^	2011	Belgium	70	B	25–74	322	9 and 5	Self-administered*‖
Bowen *et al.* ^(53)^	2012	India	530	B	ND	184	Open format§	Interview*†
Dehghan *et al.* ^(37)^	2012	Argentina	156	B	52.7 (9.5)	96	9	Interview
Park *et al.* ^(58)^	2012	Corea	288	B	30–66	112	ND	Both*‖
Macedo *et al.* ^(52)^	2013	Mexico	97	B	18–71	162	9	Interview
Babić *et al.* ^(24)^	2014	Croatia	68	B	23–57	101	7	Self-administered*‖
Gunes *et al.* ^(70)^	2015	Turkey	120	B	30–70	229	9	Interview*‖
Denova *et al.* ^(45)^	2016	Mexico	230	B	41.4 (12.2)	140	ND	Interview**
Jayawardena *et al.* ^(56)^	2016	Sri Lanka	77	B	46.5 (8.3)	85	Open format§	Interview*‖
Knudsen *et al.* ^(15)^	2016	Denmark	97	M	20–42	220	ND	Self-administered*‖**
Gazan *et al.* ^(29)^	2017	France	1863	B	18–79	94	ND	ND
Sanjeevi *et al.* ^(44)^	2017	USA	70	B	36.0 (0.8)	95	10	Interview
Whitton *et al.* ^(62)^	2017	Singapore	161	B	44.0 (14.0)	163	Open format§	Interview*
Yuan *et al.* ^(43)^	2017	USA	632	B	45–80	152	9	Self-administered
Bijani *et al.* ^(55)^	2018	Iran	200	B	68.1 (6.5)	138	Number of times daily, weekly and/or monthly	Interview*‖
Zack *et al.* ^(69)^	2018	Tanzania	317	B	52 [IQR 45, 60]	179	9	Interview††
Aoun *et al.* ^(63)^	2019	Lebanon	114	B	18–60	157	4	Interview*‡
Beck *et al.* ^(68)^	2019	New Zealand	110	W	16–45	220	9	Self-administered

IQR, interquartile range; W, women; B, both; M, men; ND, not described.

*Visual support material was used.

†Household or utensil-based measurements.

‡Food models and photographs.

§Times per day, week, month, never, etc., with non-specific ranges.

‖Photographs.

¶Food models.

**Electronic format.

††Black and white plates and food portions drawings.

The study with the fewest participants was that by Nath and Huffman^(38)^, which was conducted on twenty Cuban immigrants to the USA. The largest sample, found in a study from France, consisted of 1863 participants^(29)^. No justification was found for the sample sizes used in the validations.

More than half of the studies (81·1 %) included men and women^(12–14,16,17,19–22,24–28,30,32–34,36–38,40,43,45,48,49,51–54,56–59,61,64,66,67,70)^; 13 (24·5 %) had exclusively women participants^(5,15,23,31,35,42,44,47,50,60,62,65,68)^; three studies (5·7 %) had only men^(18,39,46)^ and one (1·9 %) did not mention the sex of participants^(41)^.

Of the total number of articles, forty-three reported minimum and maximum ages. Among them, twenty-two had minimum ages in the 18–25 range^(5,12,13,15–17,20,24,28,29,33–35,40,41,44,48,51,52,63–65)^, while three articles reported minimum ages of 13^(22)^, 16^(68)^ and 17 years^(54)^. The majority of papers (31 of 43) reported maximum ages between 42 and 75 years^(5,12–16,20,22–24,27,28,33,34,36,39,40,42,44,46,48,50,52,54,58,60,61,63,64,68,70)^. The maximum age was 100 years^(49)^.

### Quality of the studies

Most studies met at least half of the risk of bias and quality criteria that were assessed, and nine studies met less than half of the established criteria. The studies that met most of the criteria were Klipstein *et al.* ^(14)^, Chen *et al.* ^(54)^, Ocké *et al.* ^(20)^, Gunes *et al.* ^(70)^ and Whitton *et al.* ^(62)^, in which twelve of the fourteen criteria were met.

An analysis of all risk of bias and quality factors shows that the least frequently reported criterion in these studies is the sample size (five of sixty articles). Another aspect that should be improved is the representativeness of the sample, since about half of the studies have volunteer subjects (twenty-seven of sixty), and four studies do not explain how sample selection was carried out. Furthermore, twelve studies do not describe the eligibility criteria for subjects and/or the selection methods used, and twenty-four do so incompletely; twenty-five contain incomplete descriptions of locations, dates, subject recruitment periods and other related information. Finally, twenty-one studies do not describe the reasons for follow-up losses, a factor that may compromise their quality (see Supplemental Table 1a and Table 1b in the supplementary material).

### Characteristics of the semiquantitative FFQ

Table 2 also shows the number of items in each SFFQ. The lowest number of items was thirty-nine in two studies^(22,54)^, while the highest was 322^(28)^. Two articles omitted the number of items studied^(21,35)^.

Regarding the number of response categories for intake frequency, 21 studies (35 %) used nine categories^(13,17,18,31,32,35,37–39,42,43,47,50–52,61,65,67–70)^. Some studies included non-specific response options (Van Dongen *et al*.^(28)^ had 5–9 response categories depending on food group section; Fidanza^(33)^ had three categories combined with a ‘number of times’ frequency). Other articles^(1,12,14,16,20,22,23,33,34,53–56,59,62)^ included direct questions about intake frequency (options: never/rarely; number of times per month/week/day). Also, eleven did not describe such data^(5,15,19,21,25–27,29,41,45,58,64)^.

Several studies used visual support tools to enable participants to easily identify food intake amounts: seventeen studies mentioned the use of photographs^(12,15–17,20,24,25,28,33,41,49,55,56,58,61,66,70)^; four used household measures^(13,53,54,59)^; two used photographs and food models as visual aids^(27,63)^; two SFFQ used food models^(48,50)^; one used drawing of plates and different portion sizes^(69)^ and one mentioned using visual aids but it did not specify which ones^(62)^. The other articles did not say whether visual support for answering the SFFQ was provided^(1,5,14,18,19,21–23,26,29–32,34–39,42–47,51,52,57,60,64,65,67,68)^.

Most SFFQ were self-administered (thirty-two of sixty). Of these, three were sent to participants as electronic forms^(15,60,65)^; twenty-one questionnaires were administered through interviews^(1,17,22,25,37,42,44,45,48–50,52–56,59,61–63,69,70)^; two were administered combining interview and self-administered formats^(14,58)^; four did not mention the way the questionnaire was administered^(18,29,38,57)^.

### Validity of the analysed semiquantitative FFQ

Table 3 shows the main results of the validation analyses of the reviewed studies: unit of analysis (nutrients, energy and/or food groups), reference method, types of correlation coefficients used with minimum and maximum values, the time interval between the SFFQ and the comparison method, and the time interval between repeated assessments of reference method. Table 4 shows the specific correlation coefficients for energy and macronutrients.

**Table 3:** Validity of the analysed semiquantitative FFQ (SFFQ), ordered chronologically

Reference	Reference method for validation	No. of days of the reference method	Intervals between SFFQ and reference method	Intervals between the reference method	Unit of analysis	Unadjusted correlation coefficients (minimum–maximum values)	Adjusted correlation coefficients* (minimum–maximum values)
Willet *et al.* ^(42)^	FR	28	2–4 months	3 months	E&N	*r*† = 0·26–0·73	*r*† = 0·36–0·75
Willet *et al.* ^(13)^	FR	365	18 months	Each day in one year	E&N	*r*† = 0·38–0·76	*r*† = 0·21–0·68; 0·28–0·62 ‡§; 0·28–0·70*†‡§
Tjønneland *et al.* ^(27)^	WFR	14	0	2–3 weeks	E&N	*r* = M: 0·17–0·64; W: 0·26–0·53	*r* = M: 0·27–0·71; W: 0·26–0·53
Rimm *et al.* ^(39)^	FR	14	ND	6–7 months	E&N	*r*† = 0·25–0·86	*r*† = 0·28–0·87; D: 0·32–0·92
Horwath^(66)^	FR	10	2 weeks	2 months	E&N	*r* = M: 0·43–0·78; W: 0·34–0·66	ND
Longnecker *et al.* ^(51)^	FR	2–8 d	ND	Spring, summer and autumn	E&N	*r*†*‖¶* = 0·21–0·60	*r*†*‖* = 0·23–0·57; 0·24–0·57¶; D: 0·28–0·78¶
Martín *et al.* ^(5)^	FR	16	45 d	3 months	E&N	*r*† = 0·35–0·90	*r*† = 0·35–0·89; D: 0·45–0·91
Feskanich *et al.* ^(46)^	FR	14	ND	6 months	E&N	*r*† = 0·08–0·50	*r*† = 0·10–0·65; D: 0·12–0·76
Lee *et al.* ^(50)^	24HR	1	ND	ND	E&N	*r* = 0·21–0·66	ND
Porrini *et al.* ^(21)^	FR	7	ND	ND	E&N	*σ* = 0·45–0·91	ND
Ramon *et al.* ^(22)^	FR	7	8–15 d	5–11 months	E&N	*r*† = 0·17–0·54; D: 0·20–0·61	*r*† = 0·19–0·58
Rothenberg^(25)^	FR	4	ND	1 week	E&N	*r* = 0·35–0·60	ND
Fidanza *et al.* ^(33)^	WFR	7	0	0	E&N	*σ* = 0·33–0·84	ND
					FG	Displayed in tertiles	
Gnardellis *et al.* ^(12)^	24HR	12	ND	1 month	E&N	*r*†‖** = M: 0·18–0·71; D: 0·28–0·82. W: 0·04–0·58; D: −0·05–0·78	*r*†‖** = M: 0·23–0·69; W: −0·04–0·63
Grootenhuis *et al.* ^(36)^	DH	1	ND	ND	E&N	*r* = 0·36–0·77	ND
Bonifacj *et al.* ^(30)^	FR	7	ND	Each season	E&N	*r*† = 0·19–0·75	*r*† = 0·22–0·80; D: 0·25–0·80
					FG	*σ* = 0·25–0·76	ND
Friis *et al.* ^(35)^	FR	12	ND	ND	E&N	*r*†*‖* = 0·24–0·63	*r*†*‖* = 0·29–0·72; D: 0·30–0·88
Kumanyika *et al.* ^(49)^	24HR	6	0	1 month	E&N	ND	*r*†† = D: 0·24–0·73
Ocké *et al.* ^(20)^	24HR	12	0	1 month	E&N	*r*† = M: 0·26–0·83; D: 0·34–0·87. W: 0·35–0·90; D: 0·47–0·94	*r*† = M: 0·23–0·82; D: 0·29–0·85. W: 0·23–0·84; D: 0·31–0·87
Hérnandez *et al.* ^(47)^	24HR	16	0	Each season	E&N	*r*†*‖* = 0·19–0·61; D: 0·12–0·71	*r*†*‖* = 0·05–0·67
Klipstein *et al.* ^(14)^	FR	15	2 months	1–2 months	E&N	*r* = 0·47–0·89	*r*¶ = 0·39–0·83 D: 0·44–0·85
Smith *et al.* ^(67)^	WFR	4	ND	4 months	E&N	*r* = 0·19–0·68	*r* = 0·10–0·70; *σ* = 0·16–0·69
Fregapane and Asensio^(34)^	WFR	4	ND	ND	Nutrients	*σ* = 0·302–0·893; ICC = 0·199–0·875	ND
Jackson *et al.* ^(48)^	24HR	12	ND	3 months	E&N	*r*† = 0·20–0·86	*r*† = 0·17–0·85
Schröder *et al.* ^(26)^	FR	3	1–4 weeks	3 weeks	E&N	*r* = 0·17–0·61; ICC = 0·12–0·48	ND
Tokudome *et al.* ^(60)^	WFR	28	1–2 weeks	Each season	E&N	*r* = 0·17–0·68; *r*† = 0·30–0·68; *σ* = 0·26–0·63	*r*† = 0·26–0·71; D: 0·28–0·73; *σ* = 0·22–0·71
					FG	*r* = 0·14–0·69; *r*† = 0·16–0·78; *σ* = 0·29–0·68	*r*† = 0·16–0·75; D: 0·17–0·76; *σ* = 0·28–0·68
Rodríguez *et al.* ^(40)^	24HR	2–3	ND	1 month	E&N	*r*‡‡ = 0·12–0·64; D: 0·22–0·73	*r*‡‡ = 0·11–0·59; D: 0·19–0·84
					FG	*r* = 0·01–0·59	ND
Masson *et al.* ^(16)^	WFR	4	9 d	ND	E&N	ND	M: *r*† = -0·45–0·83; *σ* = -0·13–0·72. W: *r*† = 0·37–0·86; *σ* = -0·04–0·79
Moreira *et al.* ^(17)^	FR	4	ND	0	E&N	*r* = 0·21–0·73	*r* = 0·20–0·75
Chen *et al.* ^(54)^	FR	14	76 d	6–9 months	E&N	*r*† = USDA: 0·06–0·32; IND: 0·01–0·27	*r*† = USDA: 0·03–0·38; D: 0·05–0·70; IND: 0·01–0·32; D: 0·08–0·56
					FG	*r*† = 0·08–0·43	*r*† = 0·09–0·39; D: 0·19–0·78
Ke *et al.* ^(57)^	WFR	3	1 week	ND	E&N	*r* = 0·16–0·52; *r*† = 0·17–0·65; *σ* = 0·17–0·70	*r* = 0·12–0·58; *r*† = 0·20–0·63; *σ* = 0·19–0·67
					FG	*r* = 0·23–0·41; *r*† = 0·30–0·67; *σ* = 0·30–0·46	*r* = 0·31–0·53; *r*† = 0·30–0·69; *σ* = 0·31–0·57
Nath and Huffman^(38)^	FR	3	ND	ND	E&N	*r* = −0·18–0·71	*r* = -0·34–0·55
Roddam *et al.* ^(23)^	FR	7	3 months	0	E&N	*r* = 0·16–0·75	*r* = 0·12–0·75
Shatenstein *et al.* ^(41)^	24HR	4	ND	ND	E&N	*σ* = 0·30–0·57; M: 0·27–0·62; W: 0·08–0·62	ND
					FG	*σ* = 0·32–0·45	ND
Dumartheray *et al.* ^(31)^	WFR	4	ND	ND	E&N	*r* = 0·209–0·550; *r*† = 0·138–0·583	*r* = 0·011–0·631; *r*† = -0·062–0·643
Sudha *et al.* ^(59)^	24HR	6	ND	2 months	E&N	*r*† = 0·22–0·72	*r*† = 0·20–0·60; D: 0·22–0·67
Nöthlings *et al.* ^(19)^	24HR	2	ND	ND	E&N	*r*§§ = M: 0·13–0·70; D: 0·13–0·70. W: 0·30–0·97; D: 0·30–0·97. *r*‖‖ = M: 0·11–0·70; D: 0·11–0·70. W: 0·32–0·86; D: 0·13–0·47	*r*§§ = D: M: 0·19–1·00; W: 0·18–0·99. *r*‖‖ = D: M: 0·16–0·88; W: 0·25–1·00
					FG	*r*§§ = M: 0·15–0·59; D: 0·29–0·98. W: 0·15–0·61; D: 0·22–0·79. *r*‖‖ = M: 0·14–0·62; D: 0·28–0·98. W: 0·15–0·60; D: 0·22–0·80	*r*§§ = M: 0·29–0·98; W: 0·22–0·79. *r*‖‖ = M: 0·28–0·98; W: 0·22–0·80
Mullie *et al.* ^(18)^	FR	4	ND	2 weeks	E&N	ND	*r*† = 0·01–0·52
Barret and Gibson^(64)^	FR	28	0	3 months	E&N	ND	*σ* = 0·239- 0·810
Fernández *et al.* ^(32)^	FR	12	1–2 months	3 months	E&N	*r* = 0·24–0·61; ICC = 0·37–0·80	*r* = 0·24–0·65; ICC = 0·40–0·78
					FG	ND	*r* = 0·37–0·72; ICC = 0·40–0·84
Yang *et al.* ^(61)^	WFR	9	ND	Each season	E&N	ND	*r*‡ = 0·07–0·41‡; D: 0·08–0·72; r*‡ = 0·08–0·37
Fayet *et al.* ^(65)^	24HR	3	ND	2 weeks	E&N	ND	*r*† = 0·01–0·92; D: 0·41–0·99
van Dongen *et al.* ^(28)^	24HR	5	ND	2–3 months	E&N	*r* = M: 0·21–0·92; W: −0·02–0·84	ND
Bowen *et al.* ^(53)^	24HR	3	At least one week	1–2 months	E&N	*σ*† = 0·42–0·69	*σ*† = 0·43–0·52; D: 0·57–0·87
					FG	*σ*† =0·25–0·72	*σ*† = 0·24–0·59
Dehghan *et al.* ^(37)^	24HR	U: 8 24HR; R: 3 24HR	0	3–4 months	E&N	*r*† = U: 0·20–0·47; R: 0·11–0·4 l; *r*†**= D: U: 0·33–0·62; R: 0·35–0·90	ND
Park *et al.* ^(58)^	FR	3	ND	Each season	E&N	*σ* = 0·24–0·42; M: 0·06–0·41; W: 0·20–0·45	ND
					FG	*σ* = 0·15- 0·72; M: 0·21–0·61; W: 0·09–0·62	ND
Macedo *et al.* ^(52)^	FR	9	1 month	3 months	Nutrients	*r*† = 0·12–0·58; ICC† = D: 0·19–0·73	*r*† = 0·09–0·62; ICC† = D: 0·16–0·77
					FG	*r*† = 0·21–0·71; ICC† = D: 0·35–0·84	ND
Babić *et al.* ^(24)^	24HR	3	ND	Autumn, winter and spring	E&N	*r*‡ = 0·098–0·482	*r*‡ = 0·096–0·471
Gunes *et al.* ^(70)^	24HR	4	15 d	Each season	E&N	*r*† = 0·060–0·468; D: 0·107–0·655	*r*† = 0·025–0·534
					FG	*r*† = 0·190–0·630; D: 0·295–0·760	*r*† = 0·176–0·611
Denova *et al.* ^(45)^	24HR	2	0	2–3 d	E&N	*r*† = 0·26–0·49	*r*† = 0·20–0·52; D: 0·30–0·61
Jayawardena *et al.* ^(56)^	WFR	7	ND	ND	E&N	*r* = 0·09–0·52	ND
Knudsen *et al.* ^(15)^	FR	4	1–3 d	ND	E&N	ND	*r* = 0·08–0·63; D: 0·13–0·93
					FG	ND	*r* = 0·17–0·61; D: 0·25–0·75
Gazan *et al.* ^(29)^	FR	7	ND	0	E&N	*σ* = 0·66–0·90	*σ* = 0·56–0·90
					FG	*σ* = 0·82–1·00	*σ* = 0·82–1·00
Sanjeevi *et al.* ^(44)^	FR	3	ND	ND	E&N	*r*‡‡ = 0·36–0·70; D: 0·39–0·76 *r*¶¶ = 0·37–0·70; D: 0·40–0·76	ND
Whitton *et al.* ^(62)^	24HR	2	1 month	1–4 months	E&N	*r*†*‖* = 0·10–0·51	*r*†‖*** = 0·02–0·47; D: 0·02–0·64
Yuan *et al.* ^(43)^	FR	1–4	1–2 months	6 months	E&N	*σ* = 0·28–0·86; ICC = 0·23–0·86	*σ* = 0·36–0·77; 0·37–0·86 †††; D: 0·31–0·84
	24HR	7		Each season	E&N	*σ* = 0·23–0·75; ICC = 0·08–0·50	*σ* = 0·15–0·70; 0·20–0·75 †††; D: 0·29–0·77
Bijani *et al.* ^(55)^	24HR	2	ND	ND	E&N	*r* = M: −0·38–0·53; W: −0·01–0·71	ND
					FG	*r* = M: 0·06–0·62; W: −0·01–0·60	ND
Zack *et al.* ^(69)^	24HR	2	0	3 d	E&N	Rosner rank correlation coefficient= −0·04–0·26.	Rosner rank correlation coefficient= −0·2–0·26; D: −0·03–0·41 ICC = 0·09–0·38
					FG	Rosner rank correlation coefficient= 0·00–0·42; D: 0·00–0·51. ICC = 0·12–0·58	ND
Aoun *et al.* ^(63)^	24HR	3	1 week	1–2 d	E&N	0·904–0·998‡‡‡	0·783–0·998‡‡‡
					FG	0·906–1·000	ND
Beck *et al.* ^(68)^	WFR	4	ND	0	E&N	*σ* = 0·11–0·59	*σ* = 0·23–0·67

FR, food record; E&N, energy and nutrients; *r*, Pearson correlation coefficient; WFR, weighed food record; M, men; W, women; D, de-attenuated; 24HR, 24-hour recall; ND, not described; σ, Spearman’s rank correlation coefficient; FG, food groups; DH, diet history; ICC, intraclass correlation coefficient; USDA, Nutrient database from United States Department of Agriculture; IND, Indian Nutrient Database; U, Urban; R, Rural.

*Adjusted by energy, if not otherwise stated.

†With logarithmic scale transformation.

‡Adjusted only by sex.

§Adjusted only by age.

‖Coefficients from the second SFFQ reported.

¶Adjusted by energy, sex and age.

**It is not clear whether the de-attenuated coefficient was for crude or energy-adjusted values.

††Adjusted only by sex and age.

‡‡With logarithmic scale transformation for some nutrients.

§§Fitted portion size.

‖‖Predefined portion size.

¶¶Assuming uniform intake of multiple foods in a line in the FFQ.

***Adjusted for ethnicity, age and sex.

†††Energy density method (divides the nutrient portion by total energy intake).

‡‡‡Pearson o Spearman’s was used depending on normality distribution.

**Table 4:** Results of energy and macronutrient validity for the analysed studies, ordered chronologically

Reference	Correlation coefficient	Energy (kcal)		Protein (g)		Total fat (g)		Carbohydrates (g)	
		Unadjusted	Adjusted	Unadjusted	Adjusted*	Unadjusted	Adjusted*	Unadjusted	Adjusted*
Willet *et al.* ^(42)^	Pearson†	ND	–	0·33	0·47	0·39	0·53	0·53	0·45
Willet *et al.* ^(13)^	Pearson†	0·67	0·37‡	0·6	0·43; 0·46‡; 0·53 §;	0·76	0·51; 0·57‡; 0·59§	0·6	0·51; 0·37‡; 0·55§
Tjønneland *et al.* ^(27)^	Pearson	M: 0·40; W: 0·23	–	M: 0·41; W: 0·14	M: 0·52; W: 0·26	M: 0·54; W: 0·27	M: 0·67; W: 0·48	M: 0·44; W: 0·34	M: 0·40; W: 0·47
Rimm *et al.* ^(39)^	Pearson†	0·40; D: 0·43	–	0·25	0·38; D: 0·44	0·52	0·61; D: 0·67	0·48	0·69; D:0·73
Horwath^(66)^	Pearson	M: 0·69; W: 0·46	–	M: 0·78; W: 0·37	ND	M: 0·63; W: 0·52	ND	M: 0·63; W: 0·41	ND
Longnecker *et al.* ^(51)^	Pearson†	0·38	–	0·34	0·29; 0·33 §; D: 0·45 §	0·34	0·44; 0·43 §; D: 0·58 §	0·5	0·53; 0·51 §; D: 0·62 §
Martín *et al.* ^(5)^	Pearson†	0·54; D: 0·57	–	0·52	0·44; D: 0·56	0·41	0·41; D: 0·47	0·44	0·42; D: 0·46
Feskanich *et al.* ^(46)^	Pearson†	0·28; D: 0·31	–	0·17	0·11; D: 0·13	0·42	0·50; D: 0·57	0·27	0·62; D: 0·69
Lee *et al.* ^(50)^	Pearson	0·50	–	0·56	ND	0·21	ND	0·37	ND
Porrini *et al.* ^(21)^	Pearson, Spearman‖	0·76	–	0·65; Bch: 0·71	ND	0·77	ND	0·49	ND
Ramon *et al.* ^(22)^	Pearson†	0·32; D: 0·59	–	0·41; D: 0·58	0·56	0·36; D: 50·2	0·48	0·34; D: 0·59	0·54
Rothenberg^(25)^	Pearson	0·66	–	0·5	ND	0·6	ND	0·56	ND
Fidanza *et al.* ^(33)^	Spearman	0·53	–	0·48	ND	0·59	ND	0·36	ND
Gnardellis *et al.* ^(12)^	Pearson†¶**	M: 0·43; D: 0·45 W: 0·32; D: 0·34	–	M: 0·51; W: 0·34	D: M: 0·50; W: 0·29	ND	ND	ND	ND
Grootenhuis *et al.* ^(36)^	Pearson	0·72	–	0·69	ND	0·7	ND	0·72	ND
Bonifacj *et al.* ^(30)^	Pearson†	0·40; D: 0·42	–	0·29	0·22; D: 0·31	0·39	0·23; D: 0·44	0·25	0·33; D: 0·27
Friis *et al.* ^(35)^	Pearson†¶	0·27; D: 0·30	–	0·28	0·41; D: 0·52	0·38	0·49; D: 0·56	0·34	0·56; D: 0·63
Kumanyika *et al.* ^(49)^	Pearson	ND	D: 0·50‡	ND	D:0·44‡	ND	D:0·67‡	ND	D:0·43‡
Ocké *et al.* ^(20)^	Pearson†	M: 0·71; D: 0·77. W: 0·58; D: 0·62	–	M: 0·61; D: 0·68. W: 0·51; D: 0·56	M: 0·62; D: 0·71. W: 0·69; D: 0·67	M: 0·69; D: 0·74. W: 0·58; D: 0. 63	M: 0·57; D: 0·61. W: 0·57; D: 0·63	M: 0·72; D: 0·75. W: 0·66; D: 0·69	M: 0·71; D: 0·74. W: 0·72; D: 0·76
Hérnandez *et al.* ^(47)^	Pearson†¶	0·50; D: 0·52	–	0·43; D: 0·32	0·23	0·47; D: 0·63	0·58	0·56; D: 0·57	ND
Klipstein *et al.* ^(14)^	Pearson	0·69	–	0·73	0·66 §; D: 0·63 §	0·57	0·50 §; D: 0·48 §	0·72	0·79 §; D: 0·77 §
Smith *et al.* ^(67)^	Pearson	0·41	–	0·26	0·16	0·32	0·61	0·49	0·59
	Spearman	ND	–	ND	0·16	ND	0·57	ND	0·54
Fregapane and Asensio^(34)^	Spearman	ND	–	0·328	ND	0·36	ND	0·785	ND
	ICC	ND	–	0·261	ND	0·327	ND	0·786	ND
Jackson *et al.* ^(48)^	Pearson†	0·60	–	0·45	0·44	0·53	0·48	0·61	0·55
Schröder *et al.* ^(26)^	Pearson	0·29	–	0·29	ND	0·17	ND	0·31	ND
	ICC	0·19	–	0·19	ND	0·12	ND	0·26	ND
Tokudome *et al.* ^(60)^	Pearson	0·45	–	0·40	ND	0·57	ND	0·47	ND
	Pearson†	0·46; D: 0·48	–	0·42	0·51; D: 0·53	0·57	0·46; D: 0·49	0·47	0·55; D: 0·57
	Spearman	0·42	–	0·43	0·45	0·53	0·48	0·48	0·55
Rodríguez *et al.* ^(40)^	Pearson	0·64; D: 0·72	–	0·53; D: 0·64	0·17; D: 0·22	0·63; D: 0·73	0·56; D: 0·66	0·63†; D: 0·71†	0·59†; D: 0·70†
Masson *et al.* ^(16)^	Pearson†	M: 0·35; W: 0·40	–	ND	M: 0·53; W: 0·51	ND	M: 0·54; W: 0·83	ND	ND
	Spearman	M: 0·24; W: 0·39	–	ND	M: 0·25; W: 0·43	ND	M: 0·42; W: 0·64	ND	ND
Moreira *et al.* ^(17)^	Pearson	M: 0·45; W: 0·46	–	M: 0·46; W: 0·41	M: 0·38; W: 0·37†	M: 0·27; W: 0·51	M: 0·58; W: 0·53†	M: 0·57; W: 0·44	M: 0·39; W: 0·43†
Chen *et al.* ^(54)^	Pearson†	USDA: 0·12; D: 0·17; IND: 0·11; D: 0·14	–	USDA: 0·16; IND: 0·17	USDA: 0·30, D: 0·53; IND: 0·29, D: 0·52	USDA: 0·24; IND: 0·27	USDA: 0·35, D: 0·70; IND: 0·29, D: 0·56	USDA: 0·13; IND: 0·11	USDA: 0·26, D: 0·35; IND: 0·32, D: 0·53
Ke *et al.* ^(57)^	Pearson	0·26	–	0·25	0·24	0·32	0·34	0·27	0·41
	Pearson†	0·29	–	0·32	0·33	0·34	0·32	0·41	0·40
	Spearman	0·31	–	0·30	0·40	0·39	0·35	0·27	0·42
Nath and Huffman^(38)^	Pearson	0·67	–	0·14	-0·34	0·59	0·00	0·42	0·55
Roddam *et al.* ^(23)^	Pearson	0·29	–	0·36	0·31	0·34	0·48	0·49	0·64
Shatenstein *et al.* ^(41)^	Spearman	0·57; M: 0·56; W: 0·45	–	0·48; M: 0·12; W: 0·48	ND	0·57; M: 0·47; W: 0·53	ND	0·43; M: 0·62; W: 0·32	ND
Dumartheray *et al.* ^(31)^	Pearson	0·43; 0·40†	–	0·51; 0·45†	0·46; 0·46†	0·36; 0·33†	0·46; 0·41†	0·43; 0·44†	0·62; 0·53†
Sudha *et al.* ^(59)^	Pearson†	0·48; D: 0·52	–	0·52	0·37; D: 0·43	0·32	0·47; D: 0·53	0·65	0·60; D: 0·67
Nöthlings *et al.* ^(19)^	Pearson	0·29††‡‡; 0·27††§§	–	0·19††‡‡; 0·19††§§	0·33††‡‡; 0·36††§§	0·27††‡‡; 0·27††§§	0·42††‡‡; 0·53††§§	0·37††‡‡; 0·35††§§	0·48††‡‡; 0·55††§§
Mullie *et al.* ^(18)^	Pearson†	0·52	–	ND	0·29	ND	0·22	ND	0·32
Barret and Gibson^(64)^	Spearman	ND	–	ND	0·547	ND	0·488	ND	0·541
Fernández *et al.* ^(32)^	Pearson	0·36	–	0·28	0·40	0·47	0·46	0·38	0·56
	ICC	0·53	–	0·44	0·55	0·63	0·62	0·55	0·55
Yang *et al.* ^(61)^	Pearson‖‖	ND	0·31–0·36 ¶¶; D: 0·36–0·43 ¶¶	ND	0·30–0·34 ¶¶; D: 0·38–0·43 ¶¶; 0·14–0·28*¶¶	ND	0·21–0·31¶¶; 0·14–0·28¶¶; 0·23–0·29*¶¶	ND	0·38–0·41¶¶; 0·23–0·29 ¶¶; 0·22–0·27*¶¶
Fayet *et al.* ^(65)^	Pearson†	0·40	–	ND	0·92 ***; D: 0·99 ***	ND	ND	ND	D: 0·40
van Dongen *et al.* ^(28)^	Pearson	M: 0·53; W: 0·47	–	M: 0·55; W: 0·42	ND	M: 0·26; W: 0·29	ND	M: 0·76; W: 0·63	ND
Bowen *et al.* ^(53)^	Spearman†	0·62	0·71	0·61	0·50; D: 0·87	0·42	0·52; D: 0·57	0·69	0·52; D: 0·76
Dehghan *et al.* ^(37)^	Pearson†**	U: 0·44; R: 0·35; D: U: 0·51; R: 0·53	–	U: 0·26; R: 0·33; D: U: 0·39; R: 0·53	ND**	U: 0·38; R: 0·34; D: U: 0·50; R: ND	ND**	U: 0·47; R: 0·33; D^;^ U: 0·57; R: 0·46	ND**
Park *et al.* ^(58)^	Spearman	0·40; M: 0·21; W: 0·32	–	0·32; M: 0·23; W: 0·29	ND	0·38; M: 0·34; W: 0·31	ND	0·24; M: 0·06; W: 0·27	ND
Macedo *et al.* ^(52)^	Pearson†	0·50	–	0·41	0·38	0·48	0·10	0·45	0·16
	ICC†	0·67	–	D: 0·58	D: 0·56	D: 0·64	D: 0·18	D: 0·60	D: 0·27
Babić *et al.* ^(24)^	Pearson	0·41	0·34 ¶¶	0·29	0·32 ¶¶	0·4	0·37¶¶	0·48	0·42¶¶
Gunes *et al.* ^(70)^	Pearson†	0·47; D: 0·66	–	0·38; D: 0·49	0·25	0·43; D: 0·64	0·35	0·47; D: 0·63	0·53
Denova *et al.* ^(45)^	Pearson†	0·45; D: 0·53	–	0·37	0·20; D: 0·33	0·44	0·43; D: 0·51	0·43	0·37; D: 0·49
Jayawardena *et al.* ^(56)^	Pearson	0·39	–	0·26	ND	0·17	ND	0·47	ND
Knudsen *et al.* ^(15)^	Pearson	0·29; D: 0·33	–	ND	0·49, D: 0·56	ND	0·56, D: 0·63	ND	0·63, D: 0·70
Gazan *et al.* ^(29)^	Spearman	0·87	–	0·82	0·81	0·86	0·86	0·87	0·87
Sanjeevi *et al.* ^(44)^	Pearson†††	0·70, D: 0·74	–	0·53, D: 0·57	ND	ND	ND	0·65, D: 0·70	ND
Whitton *et al.* ^(62)^	Pearson†¶	0·11	0·02; D: 0·02	0·36 ***	0·28***‡‡‡; D: 0·45***‡‡‡	0·33 ***	0·30***‡‡‡; D: 0·39***‡‡‡	0·25 ***	0·15***‡‡‡; D: 0·20***‡‡‡
Yuan *et al.* ^(43)^	Spearman (FR)¶	0·28; D: 0·31	–	0·33	0·48; 0·48§§§ D: 0·54	0·36	0·59; 0·59§§§;D: 0·67	0·41	0·65; 0·66§§§; D: 0·69
	Spearman (24HR)¶	0·30; D: 0·40	–	0·30	0·37; 0·38§§§; D: 0·54	0·34	0·55; 0·55§§§; D: 0·76	0·44	0·58; 0·58§§§; D: 0·73
Bijani *et al.* ^(55)^	Pearson	M: 0·53; W: 0·71	–	M: 0·39; W: 0·55	ND	M: 0·49; W: 0·46	ND	M: 0·52; W: 0·69	ND
Zack *et al.* ^(69)^	Rosner†	0·12	–	0·20	0·11; D: 0·22	0·21	0·09; D: 0·22	0·07	0·16; D: 0·25
	ICC	0·38	–	ND	0·18	ND	0·20	ND	0·26
Aoun *et al.* ^(63)^	Pearson, Spearman‖	0·998	–	0·996	0·989	0·996	0·986	0·996	0·989
Beck *et al.* ^(68)^	Spearman	0·32	–	0·32	0·49	0·50	0·54	0·38	0·49

ND, not described; M, men; W, women; D, de-attenuated; Bch, biochemical analyses; ICC, intraclass correlation coefficient; USDA, Nutrient database from United States Department of Agriculture; IND, Indian Nutrient Database; U, urban; R, Rural; FR, food record; 24HR, 24-hour recall.

*Adjusted by energy, if not otherwise stated.

†With logarithmic scale transformation.

‡Adjusted by sex and age.

§Adjusted by energy–age–sex.

‖Pearson’s correlation was used for variables with normal distributions and Spearman’s for non-parametrically distributed variables.

¶Coefficients from the second SFFQ are reported.

**It is not clear whether the coefficients were for raw or adjusted values.

††Averaged values for male and female subjects.

‡‡Fitted portion size.

§§Predefined portion size.

‖‖Displayed as a range because correlation coefficients are shown for each season of the year.

¶¶Adjusted by sex.

***Presented as percentage of energy.

†††With logarithmic scale transformation for some nutrients.

‡‡‡Adjusted for ethnicity, age and sex.

§§§Energy density method (divides the nutrient portion by total energy intake).

All studies analysed the validity of SFFQ concerning nutrients and energy, except for two that presented no energy data^(34,52)^. Eighteen studies focused on ‘food groups’ as the unit of analysis^(1,15,19,29,30,32,33,41,52–55,57,58,60,63,69,70)^ (Table 3). Regarding the number of elements and/or nutrients analysed, the lowest number reported was six: energy, proteins, carbohydrates, lipids, SFA and fibre^(53)^. Of the total number of studies, 23 (38·3 %) analysed 10–20 elements including energy^(1,5,12,13,18–22,26–28,33,37,38,42,45,48,50,58,62,63,65)^, while 22 analysed 20–30^(15,16,23,24,30,35,39,41,44,46,47,51,52,54–57,59,61,68–70)^, although the same nutrients were not always counted in each study. The study which analysed the greatest number of elements (including energy) was Yuan *et al.* with 45^(43)^ (data not shown).

Of all the articles, thirty-seven were validated using food records as the standard of comparison^(5,13–18,21–23,25–27,29–35,38,39,42–44,46,51,52,54,56–58,60,61,64,66,67)^, and among these, eleven employed food weighing^(16,27,31,33,34,56,57,60,61,67,68)^. The fewest number of days reported when food records were used was three^(26,38,44,57,58)^, while the greatest was 365^(13)^. One study did not indicate a specific number of days during which records were used, reporting instead a 2–8 d range^(51)^. A total of twenty-three articles were validated by means of several 24-h recalls with intervals ranging from 1 to 16 d^(12,19,20,24,28,37,40,41,43,45,47–50,53,55,59,62,63,65,69,70)^. Two of these articles lacked established ranges^(37,40)^, and another reported different numbers of days over which 24-h recalls were administered to the same sample^(43)^ (Table 3).

Regarding the validity analysis, forty-one of the studies used Pearson’s correlation coefficients^(5,12–15,17–20,22–25,27,28,30,31,35–40,42,44–51,54–56,59,61,62,65,66,70)^, while nine used Spearman’s^(21,29,33,41,43,53,58,64,68)^. Both Pearson’s and Spearman’s coefficients were used in four articles^(16,57,60,67)^. Three articles used intraclass and Pearson’s correlation coefficients^(26,32,52)^, one used intraclass and Spearman’s^(34)^, another used Rosner’s and intraclass correlation coefficients^(69)^, while other only mentioned using Pearson or Spearman’s correlation coefficient depending variables normality^(63)^ (Table 3).

Most correlation coefficients were adjusted by energy^(5,12,15–20,22,23,27–33,35,37–40,42,43,45–48,51–54,57,59,60,63–65,67–70)^. One study adjusted the coefficients by the participants’ sex^(24)^, another adjusted them by sex and age^(49)^, other was adjusted by ethnicity, age and sex^(62)^ and an additional study adjusted them by sex, energy and age^(13)^. Twelve studies did not perform this coefficient adjustment^(21,25,26,34,36,41,44,50,55,56,58,66)^ (Table 3).

In general, correlation coefficients for energy and nutrients ranged from −0·45^(63)^ to 1·00^(19)^. Crude correlation coefficients ranged from −0·38^(55)^ to 0·998^(63)^; crude and de-attenuated correlation coefficients ranged from −0·05^(12)^ to 0·98^(19)^; adjusted values ranged from −0·45^(16)^ to 0·998^(63)^; and adjusted and de-attenuated values ranged from −0·03^(69)^ to 1·00^(19)^ (Table 3). For good groups, correlation coefficients ranged from −0·01^(55)^ to 1·00^(63)^ (crude values).

In the case of correlation coefficients for energy and macronutrients (Table 4), we found that the lowest correlation coefficient was −0·34 for proteins^(38)^ (Pearson’s, adjusted), while the highest were 0·99^(63,65)^ (Pearson’s, adjusted and crudes) for proteins^(63,65)^, also for carbohydrates^(63)^ and total fat^(63)^ (Pearson’s, crude). In the case of energy, correlation coefficient range was between 0·02^(62)^ (Pearson’s, adjusted) and 0·99^(63)^ (Pearson’s, or Spearman’s, crude).

Another important issue is related to the time interval between the repeated assessment of the reference method, and the time interval between the SFFQ and the comparison method. In nine studies, the SFFQ and the reference method were applied at the same time^(20,27,33,37,45,47,49,64,69)^; one study^(15)^ gave less than a week; eight studies gave an interval of 1–2 weeks^(16,22,53,57,60,63,66,70)^ and other eight gave an interval of 1–2 months^(1,5,13,14,32,43,52,62)^; four gave more than 2 months between methods^(23,42,46,54)^, while three studies gave different time intervals^(12,26,51)^. It is important to notice that the remaining twenty-seven studies do not report the time frame between SFFQ and reference method^(17–19,21,24,25,28–31,34–36,38,39,41,44,48,50,55,56,58,59,61,65,67,68)^.

In the case of repeated assessments of the reference method, timelines were varied, but the most frequent was every 3 months or in each season^(5,30,32,37,42,47,48,51,52,58,60,61,64,70)^ or this was not described in the paper^(15,16,19,21,31,34–36,38,41,44,50,55–57)^.

It is important to notice that the nutrients mentioned most frequently across studies, in addition to energy and macronutrients, were cholesterol, SFA, PUFA, fibre, vitamin C, Ca and Fe. PUFA had the lowest coefficient, which was −0·10^(55)^ (Pearson’s, crude), while vitamin C showed the highest coefficient value of 0·98^(63)^ (Pearson’s, or Spearman’s, crude). These data are detailed in Supplemental Table 2 in the online supplementary material.

### Reproducibility of the analysed semiquantitative FFQ

Of the sixty validation articles analysed, twenty-five included reproducibility analyses^(5,12,14,18,20,23,26,31,32,35,37,39,42–44,47,48,51,52,58,59,62–64,67)^. Table 5 shows their characteristics.

**Table 5:** Reproducibility of the analysed semiquantitative FFQ (SFFQ), ordered chronologically

Reference	Separation between SFFQ	Correlations coefficients (minimum and maximum values)*		Intraclass correlation coefficients (minimum and maximum values)		Classification of reproducibility
		Unadjusted	Energy adjusted	Unadjusted	Energy adjusted	
Willet *et al.* ^(42)^	1 year	*r* = 0·52–0·71	ND	*r* = 0·49–0·71	ND	ND
Rimm *et al.* ^(39)^	1 year	ND	ND	*r* = 0·47–0·80	*r* = 0·35–0·79	R
Longnecker *et al.* ^(51)^	6–12 months	*r* = 0·39–0·81	*r* = 0·43–0·80	ND	ND	ND
Martín *et al.* ^(5)^	1 year	*r* = 0·51–0·88	ND	ND	ND	GR
Gnardellis *et al.* ^(12)^	1 year	ND	ND	*r* = M: 0·38–0·77; W: 0·39–0·78	*r* = M: 0·31–0·75; W: 0·30–0·79	R
Friis *et al.* ^(35)^	1 year	*r* = 0·24–0·63; D: 0·30–0·88	*r* = 0·24–0·69	ND	ND	R
Ocké *et al.* ^(20)^	6 months	*r* = M: 0·78–0·94; W: 0·61–0·93	*r* = M: 0·71–0·94; W: 0·46–0·93	ND	ND	ND
	1 year	*r* = M: 0·70–0·89; W: 0·59–0·94	*r* = M: 0·64–0·89; W: 0·60–0·94			
Hérnandez *et al.* ^(47)^	1 year	ND	ND	*r* = 0·43–0·60	*r* = 0·25–0·54	R
Klipstein *et al.* ^(14)^	2 years	ND	ND	*r* = 0·49–0·88	ND	ND
Smith *et al.* ^(67)^	1 month	*r* = 0·66–0·95; *σ* = 0·67–0·96	ND	ND	ND	RA
	12–18 months	*r* = 0·32–0·83; *σ* = 0·29–0·88	ND	ND	ND	
Jackson *et al.* ^(48)^	4–8 weeks (*n* 33)	*r* = 0·49–0·94	*r* = 0·49–0·93	ND	ND	RR
	1 year (*n* 90 and *n* 123)	*r* = 0·29–0·68 (*n* 90); 0·42–0·71 (*n* 123)	*r* = 0·34–0·68 (*n* 90); 0·42–0·60 (*n* 123)	*r* = 0·42–0·69 (*n* 123)	ND	GR
Schröder *et al.* ^(26)^	Short-term	*r* = 0·60–0·95	ND	*r* = 0·52–0·94	ND	ND
Roddam *et al.* ^(23)^	1–2 years	ND	*r* = 0·25–0·87 (1 year); 0·21–0·81 (2 years)†‡	ND	ND	ER
Dumartheray *et al.* ^(31)^	1 month	*r* = 0·37–0·84; 0·44–0·90§	*r* = 0·23–0·93; 0·57–0·89 §	ND	ND	ND
	1 year	*r* = 0·16–0·66; 0·07–0·95 §	*r* = -0·02–0·83; 0·01–0·93 §	ND	ND	
Sudha *et al.* ^(59)^	1 year	*r* = 0·41–0·78	ND	ND	ND	RR
Mullie *et al.* ^(18)^	2 weeks	ND	*r* = 0·42–0·79	ND	ND	RR
Barret and Gibson^(64)^	1 year	ND	ND	*r* = 0·35–0·93	ND	R
Fernández *et al.* ^(32)^	1 year	*r* = 0·50–0·78	*r* = 0·52–0·77; 0·47–0·82¶	*r* = 0·67–0·88	*r* = 0·68–0·87; 0·63–0·90‖	GR
Dehghan *et al.* ^(37)^	1 year	*r* = U: 0·30–0·56; D: 0·32–0·60	ND	*r* = U: 0·10–0·54; R: 0·33–0·60	ND	RR
Park *et al.* ^(58)^	1 year	*σ* = 0·50–0·64; 0·41–0·82‖	ND	ND	ND	RA
Macedo *et al.* ^(52)^	1 year	*r* = 0·18–0·73; 0·27–0·77‖	*r* = 0·20–0·69	*r* = 0·30–0·85; 0·42–0·87‖	*r* = 0·34–0·82	R
Sanjeevi *et al.* ^(44)^	1 month	ND	ND	0·39–0·76	ND	RA
Whitton *et al.* ^(62)^	6 months	ND	ND	0·62–0·85§	ND	GR
Yuan *et al.* ^(43)^	1 year	ND	ND	*r* = 0·50–0·91	*r* = 0·46–0·86; 0·56–0·91¶	ND
Aoun *et al.* ^(63)^	4 months	ND	ND	0·822–0·998	ND	ER

SFFQs, Semiquantitative food-frequency questionnaires; *r*, Pearson correlation coefficient; ND, Not described; R, Reproducible; GR, Good reproducibility; M, Men; W, Women; RA, Reasonably acceptable; *σ*, Spearman’s rank correlation coefficient; RR, Relatively reproducible; ER, Excellent reproducibility; U, Urban; R, Rural.

*Pearson’s correlation coefficient, unless otherwise noted.

†Performed on a larger sample of people; not the same *n* that were validated.

‡Kappa.

§Log-transformation.

‖For food groups.

¶Energy density method (divides the nutrient portion by total energy intake).

Most articles (thirteen of twenty-five) used an interval of 1 year to assess the reproducibility of the SFFQ^(5,12,20,31,32,35,37,39,42,43,47,52,58,59,64)^. Four reported reproducibility for short- and long-term^(20,31,48,67)^; two set reproducibility for more than 1 year^(14,23)^; one mentioned a 6–12 month interval^(51)^; and others mention 6-^(62)^, 4-^(63)^ or 1-month interval^(44)^ or a less than 6-month interval^(18)^. One article did not specify the interval used, stating only that it was short-term^(26)^.

The twenty-five papers that included reproducibility analyses used correlation coefficients. Eight articles used only the Pearson’s correlation coefficient^(5,18,20,23,31,35,51,59)^; nine used only the intraclass correlation coefficient^(12,14,39,43,44,47,62–64)^, and six articles reported correlation values for both coefficients^(26,32,37,42,48,52)^. Only one article used both Pearson’s and Spearman’s correlation coefficient^(67)^; another used only Spearman’s correlation coefficient^(58)^.

Uncorrected Pearson’s and Spearman’s correlation coefficients ranged from 0·16^(31)^ to 0·96^(67)^, while the adjusted values were from −0·02^(31)^ to 0·94^(20)^. Moreover, the unadjusted intraclass correlation coefficients ranged from 0·10^(37)^ to 0·99^(63)^, while adjusted values were from 0·25^(47)^ to 0·91^(43)^.

The studies included the following reproducibility categories: ‘reasonably reproducible’^(58,67)^, ‘relatively reproducible’^(18,37,48,59)^, ‘reproducible’^(12,35,39,47,52,64)^, ‘good level of reproducibility’^(5,32,48,62)^, ‘excellent reproducibility’^(23,63)^ and ‘reasonably acceptable’^(44)^. Seven articles presented correlation coefficients (although they are not stated textually) that can be considered as acceptable or good^(14,20,26,31,42,43,51)^. The study by Jackson *et al.* ^(48)^, which assesses both short- and long-term reproducibility, was classified as ‘relatively reproducible’ and as having a ‘good level of reproducibility’.

### Other analyses

In addition to correlation coefficients, fifteen articles included Kappa analyses to assess the classification capacities of the tools^(16,23,29,40,44,45,53,57,60,63,64,67–70)^. Furthermore, Bland–Altman plots were included in twenty-one articles to assess agreement between methods^(15,17,25,28,31,32,37,40,43,45,52,53,55,56,59,63–65,68–70)^. Ten of these confirmed an overestimation of the SFFQ with respect to the reference method: six used 24-h recalls^(28,40,53,59,63,70)^ and four dietary records^(31,32,52,69)^. The remaining studies did not show systematic errors for most nutrients^(15,17,25,37,43,45,55,56,64,65,68)^. For more details, see Supplemental Table 1b in the online supplementary material.

## Discussion

The main objective of this review is to provide detailed data on the validation of SFFQ. A total of sixty SFFQ that met the selection criteria were found from several geographical regions. Europe is the region with the highest number of published studies (*n* 25)^(5,12,14–36)^, while the number of studies from Latin America^(37,40,45,47,52)^ has increased significantly in recent years. These data may indicate that the number of epidemiological assessments of diet in Latin America has probably increased recently since food-related health problems have become more prevalent in that region.

More than half of the studies (81·1 %) included male and female subjects. Some authors had specific reasons for selecting subjects of only one sex. For example, Willett *et al.* ^(42)^ selected women because their study targeted female nurses; in the case of SFFQ administered only to men^(18,39,46)^, no reasons were given for this exclusive selection, with the exception of the study on male health professionals^(39)^.

Regarding age, the broadest age ranges were reported in the studies by Gazan *et al.* ^(29)^ and Shatenstein *et al.* ^(41)^. The latter had the broadest range, with 64 years of difference between the lowest and highest values. The existence of such a broad age range in the SFFQ validation process supports the administration of this questionnaire to older adults and younger populations. Notice that it was decided to include articles with minimum ages below 18 years because the mean age was not influenced by these values^(22,54,68)^.

Significant correlations were found regarding the analyses carried out for the validation of the SFFQ in the reviewed papers. Most of the studies used Pearson’s coefficients (68 %)^(5,12–15,17–20,22–25,27,28,30,31,35–40,42,44–51,54–56,59,61,62,65,66,70)^. However, no consensus has been found in the literature concerning which statistical method is most suitable for assessing the validity of dietary tools^(16)^.

The Pearson’s (*r*) and Spearman’s (*ρ*) correlation coefficients measure the degree of linear association between two variables, the former being more suitable for normal distribution and the latter for non-normal distribution. However, neither of these coefficients provides information about the degree of agreement observed, nor about the presence of systematic differences between measurements or instruments^(71–74)^. The intraclass correlation coefficient is usually used for this purpose in reproducibility studies^(72,74,75)^. However, it has been proposed as an alternative method to evaluate the agreement observed between methods in validation studies^(32,52,76)^. Taking these observations into consideration, it is suggested that validation studies include, in addition to the mean values of each method, the Pearson’s or Spearman’s correlation coefficients and, if desired, the intraclass correlation coefficient.

Regarding the interpretation of the correlation coefficients, Landis and Koch’s^(56)^ classification for the Kappa (*κ*) coefficient has been often used for this purpose. This classification is broken down as follows: 0 = poor agreement; 0·01–0·20 = slight agreement; 0·21–0·40 = fair agreement; 0·41–0·60 = moderate agreement; 0·61–0·80 = substantial agreement; 0·81–1·00 = almost perfect agreement. Other authors have suggested the following classification: 0 = none existent; *r* < 0·3 = poor; 0·30–0·70 = moderate; *r* > 0·70 = strong^(77)^. The correlation coefficients in the reviewed studies suggest that validation may be possible, although the results of the same could vary from poor to excellent.

It should be noted that analyses adjusted by energy, sex or age may be added to the crude correlation analyses. The reason for the inclusion of these analyses is that nutritional consumption may be higher or lower depending on the dependent variable (e.g. energy). Most of the included studies adjust their analysis in accordance with energy consumption using the residual method^(5,12,15–20,22,23,27,28,30–33,35,37–40,42,43,45–48,51–54,57,59,60,64,65,67,70)^. Another common practice is to convert the correlation coefficients into logarithmic scales. This is done for two main purposes: to improve the normality of the distribution of variables and to simplify the interpretation of correlation values of 0^(45)^. Almost half of the included studies make this modification^(5,12,13,16,20,22,30,31,35,37,39,42,45–48,51–54,57,59,60,65,70)^.

In addition to the adjustment for energy, daily variations in the intake of each person (random within-person error) may affect the relationship between the actual and observed nutrient intake. A random within-person error may be due either to real variations in the intake or to intake measurement errors. This type of error tends to attenuate or decrease the regression coefficients between SFFQ and reference methods (dietary records or recalls) towards zero (decrease in the strength of the association)^(3)^, which is why many authors present de-attenuated correlation analyses based on either unadjusted^(12,19,37,40,70)^ or adjusted coefficients^(5,14,15,19,20,30,35,40,43,45,46,52–54,59–61,65,69)^.

The correlation coefficients used for the validity analyses can vary due to a range of criteria. Regarding the number of items, high variability was found among SFFQ validation studies. However, we did not formally evaluate the correlation between the number of items and validity correlations because of the different populations and the different comparison methods and nutrients that were assessed. The literature associates the more stable reliability of the SFFQ^(78)^ and higher correlation coefficients^(79)^ with their greater number of items, even though this association was not reflected in this review. However, SFFQ with greater numbers of items can be tedious and tiring to answer (which may lead to bias), and having them administered by qualified interviewers may entail a considerable investment of time and money. Ultimately, it is important to keep in mind that the number of items will depend on the purpose of the questionnaire^(3)^.

About the number of participants, variability between studies and variations in correlation coefficients were also observed. This trend is evident in the study by Nath and Huffman^(38)^ in which, probably because of the small number of participants, the coefficient ranges were −0·18 to 0·71 (unadjusted) and −0·34 to 0·55 (adjusted).

Another point of interest regarding SFFQ validation process is the way they are administered. In this review, twenty-two studies state that their questionnaires were administered by an interviewer^(1,17,22,25,37,42,44,45,48–50,52–54,56,59,61–63,68–70)^. The advantage of having questionnaires administered by qualified interviewers is the assurance that they will be completed correctly^(3,79)^. When budgetary constraints prohibit hiring specialists to administer questionnaires, self-administration may be a viable alternative. Nonetheless, while self-administration saves costs associated with having someone on hand to explain how to complete the survey, it may entail a greater risk of bias if participants are not adequately informed of the procedures they should follow^(79)^.

Some SFFQ include visual support material to facilitate the estimation of participants’ food intake^(12,13,15–17,20,24,25,27,28,33,41,48–50,53–56,58,59,61–63,66,69,70)^. However, this practice has not been clearly associated with higher correlation coefficients. Some studies^(5,12,20,23,26,28,31,32,37,42,43,48–50,52–55,58,59,64,68)^ mention purposes for which their SFFQ had been previously used, such as epidemiological studies of diabetes mellitus^(48)^ or cancer^(5,12,20,42,58)^.

For validation purposes, in addition to correlation coefficients, Kappa analyses or Bland–Altman plots are usually used. The classification capacities of the tools can be analysed by comparing, through Kappa analyses and contingency tables, the concordance or agreement within the distribution by tertiles, quartiles or quintiles. Results can be reported as an exact agreement (classified in the same category by both methods), plus or minus one category, and gross misclassification^(76)^. The main advantage of this kind of analysis is that with cross-classification, the percentages misclassified clearly illustrate the likely impact of measurement error. It has been established that 50 % of subjects correctly classified and <10 % of subjects grossly misclassified into thirds, and weighted kappa values above 0·4 are desirable for nutrients of interest^(16)^. However, this is difficult to achieve, since only six studies from the fifteen reporting kappa analyses^(1,16,29,45,63,64)^ report a median or most of subjects with a 50 % or more of correct classification, three report a correct classification between 42 and 49 %, two do not report agreement in percentage^(23,63)^ and four report a lower level of agreement^(67–70)^.

By the other hand, Bland–Altman charts graphically assess agreement between the methods, displaying the under- or over-estimate of the method to be validated and identifying the possible presence of bias in the estimate. They have the advantage of not being influenced by variations from one person to another^(76,80)^. In total, almost half of the studies reporting Bland–Altman charts (11 of 21) did not show systematic errors for most nutrients^(15,17,25,37,43,45,55,56,64,65,68)^ and the others confirmed an overestimation of the SFFQ with respect to the reference method.

Other authors have carried out other literature reviews on a SFFQ creation or validation process. For example, Cade *et al.* ^(76,79)^ conducted an electronic database search for English-language papers on the creation, validation and administration of SFFQ from 1980 to 1999, from which they published two papers: a non-systematic review^(76)^ and a semi-systematic review^(79)^. At the end of each paper, they present general recommendations regarding the design, validation and administration of SFFQ. Similarly, Wakai^(78)^ conducted another literature review to identify articles in which SFFQ were developed and/or validated exclusively for the Japanese population. The main difference between these three studies and ours is that we performed a systematic review using PRISMA statement criteria; moreover, Wakai focused only in studies for Japanese population, while we included all the published studies that met inclusion criteria, regardless of the country.

Based on the analysis of previous proposals^(3,76,80,81)^ and our observations, and taking into consideration that the essential reason to validate an instrument is to confirm that it evaluates variables adequately, we recommend taking into consideration specific elements to validate SFFQ that were designed to evaluate global food intake in adults, such as the number of items; the number and sex of participants for validation; administration of the SFFQ (interview or self-reporting); seasonal fluctuations in dietary intake; visual support material for use during questionnaire administration; number of applications of the reference method (dietary records or 24-h recalls); unit of analysis: energy, nutrients and food groups; the use of unadjusted, adjusted and de-attenuated correlation coefficients for the validity analysis (depending on whether the distribution of variables is normal); the Bland–Altman plots; a reproducibility analysis between questionnaire administrations; identification of the statistical package used to perform statistical analyses; and identification of the software used to perform the nutritional analysis.

Finally, we note that when SFFQ validation studies are conducted, validity results are not always favourable for all nutrients or food groups evaluated. Ideally, improvements should be made to these SFFQ, after which they should be revalidated. At least, the limitations of these instruments should be acknowledged, and their results should be interpreted with caution. However, we believe that following the recommendations regarding the limitations of other studies and those we have discussed here will lead to better validation results.

Among the strengths of our study are the inclusion of questionnaires produced in several geographical regions and continents, and that its search period was not limited. Hence, our results include papers that are among the oldest available in the database up to those published as late as January 2020. Furthermore, we have included articles written in English, Portuguese, French and Spanish that were found using systematic search processes. We also extracted data directly from tables to prevent data omission. The scoring system we used is intended to help researchers select those SFFQ that would be the most complete and suitable for the objectives and target populations of their studies.

However, our review also has limitations. One of these is that PubMed was the only search engine used, thus resulting in the exclusion of relevant papers not retrievable through it. We nonetheless decided to only include articles found in PubMed because it is a proven source of articles from highly regarded scientific journals. Also, it may be questionable to have included studies validated only against dietary assessment tools and not studies focused on nutrients for which there might be unbiased biomarkers such as urinary potassium, urinary nitrogen (proteins) and doubly labelled water (energy). However, biomarkers have limitations, which include the fact that recovery markers (those that refer to a measure of absolute intake per 24 h) are not available for most nutrients and may, therefore, provide limited information. Therefore, we consider that biomarkers may be more suitable in studies focused on specific nutrients and not on the general diet, as in our study.

As future perspectives, the creation and validation of new food-frequency consumption questionnaires are justified, since we are living in the personalised nutrition era. Having specific tools for diverse population groups and diverse purposes will support research and application of new knowledge. Besides, it is desirable that these questionnaires not only focus on assessing food but also processed or prepared products, to not limiting existing evidence between food/nutrition and health risks.

## Conclusions

The characteristics of and validation processes for different SFFQ can vary substantially, even within individual countries. Therefore, the composite components of SFFQ should be carefully reviewed when being selected. Having described the parameters of and results from different validations, we conclude that even in cases where all SFFQ are reported as validated, their coefficients may vary. The results of this analysis show that even in cases where correlation coefficients range from poor to excellent, validation may still be feasible provided that overall results are interpreted with caution.

## Supplementary material

For supplementary material accompanying this paper visit https://doi.org/10.1017/S1368980020001834. click here to view supplementary material

## References

[1] Rodríguez I , Fernández J , Pastor G et al. (2008) Validación de un cuestionario de frecuencia de consumo alimentario corto: reproducibilidad y validez [Validation of a short food consumption frequency questionnaire: reproducibility and validity]. Nutr Hosp 23, 242–252. 18560701

[2] Herrán O , Gamboa Delgado E & Prada G (2006) Métodos para la derivación de listas de chequeo en estudios de consumo dietario [Methods for the derivation of checklists in studies of dietary consumption]. Rev Chil Nutr 33, 488–497.

[3] Willett W (2013) Nutritional Epidemiology. 3rd ed. New York: Oxford University Press.

[4] Chinnock A (2011) Development of a food frequency questionnaire and a comparison with food records. Perspect en Nutr Humana 13, 57–69.

[5] Martin-Moreno JM , Boyle P , Gorgojo L et al. (1993) Development and validation of a food frequency questionnaire in Spain. Int J Epidemiol 22, 512–519. 8359969 10.1093/ije/22.3.512

[6] Moher D , Liberati A , Tetzlaff J et al. (2010) Preferred reporting items for systematic reviews and meta-analyses: the PRISMA statement. Int J Surg 5, 336–341. 10.1016/j.ijsu.2010.02.007 20171303

[7] Wells G , Shea B , O’Connell D et al. The Newcastle-Ottawa Scale (NOS) for assessing the quality of nonrandomised studies in meta-analyses. http://www.ohri.ca/programs/clinical_epidemiology/oxford.asp (accessed October 2019).

[8] Higgins JPT , Green S (editors). Cochrane Handbook for Systematic Reviews of Interventions Version 5.1.0. https://es.cochrane.org/sites/es.cochrane.org/files/public/uploads/Manual_Cochrane_510_reduit.pdf (accessed October 2019).

[9] von Elm E , Altman D , Egger M et al. (2007) Strengthening the reporting of observational studies in epidemiology (STROBE) statement: guidelines for reporting observational studies. BMJ 335, 806–808. 17947786 10.1136/bmj.39335.541782.AD PMC2034723

[10] Katsouyanni K , Rimm EB , Gnardellis C et al. (1997) Reproducibility and relative validity of an extensive semi-quantitative food frequency questionnaire using dietary records and biochemical markers among Greek schoolteachers. Int J Epidemiol 26, 118–127. 10.1093/ije/26.suppl_1.s118 9126540

[11] NA (2017) Reproducibility and validity of a semiquantitative food frequency questionnaire. Am J Epidemiol 185, 1109–1123. 30052738 10.1093/aje/kwx107

[12] Gnardellis C , Trichopoulou A , Katsouyanni K et al. (1995) Reproducibility and validity of an extensive semiquantitative food frequency questionnaire among Greek school teachers. Epidemiology 6, 74–77. 7888451 10.1097/00001648-199501000-00015

[13] Willett WC , Reynolds RD , Cottrell-Hoehner S et al. (1987) Validation of a semi-quantitative food frequency questionnaire: comparison with a 1-year diet record. J Am Diet Assoc 87, 43–47. 3794132

[14] Klipstein-Grobusch K , Den Breeijen JH , Goldbohm RA et al. (1998) Dietary assessment in the elderly: validation of a semiquantitative food frequency questionnaire. Eur J Clin Nutr 52, 588–596. 9725660 10.1038/sj.ejcn.1600611

[15] Knudsen VK , Hatch EE , Cueto H et al. (2016) Relative validity of a semi-quantitative, web-based FFQ used in the “Snart Forældre” cohort – a Danish study of diet and fertility. Public Health Nutr 19, 1027–1034. 26235206 10.1017/S1368980015002189 PMC7681628

[16] Masson LF , MCNeill G , Tomany JO et al. (2003) Statistical approaches for assessing the relative validity of a food-frequency questionnaire: use of correlation coefficients and the kappa statistic. Public Health Nutr 6, 313–321. 12740081 10.1079/PHN2002429

[17] Moreira P , Sampaio D & Almeida M (2003) Validity assessment of a food frequency questionnaire by comparison with a 4-day diet record. Acta Med Port 16, 412–420. 15631853

[18] Mullie P , Clarys P , Hulens M et al. (2009) Reproducibility and validity of a semiquantitative food frequency questionnaire among military men. Mil Med 8, 852–856. 10.7205/milmed-d-00-1409 19743742

[19] Nöthlings U , Hoffmann K , Bergmann M et al. (2007) Fitting portion sizes in a self-administered food frequency questionnaire. J Nutr 137, 2781–2786. 18029499 10.1093/jn/137.12.2781

[20] Ocké MC , Bueno-de-Mesquita HB , Pols MA et al. (1997) The Dutch EPIC food frequency questionnaire. II. Relative validity and reproducibility for nutrients. Int J Epidemiol 26, Suppl. 1, S49–S58. 9126533 10.1093/ije/26.suppl_1.s49

[21] Porrini M , Gentile MG & Fidanza F (1994) Validity of a self-administered, semiquantitative food frequency questionnaire. Bibl Nutr Dieta 41–44. 7695584 10.1159/000423779

[22] Ramón J , Micaló T , Benítez D et al. (1994) Dietary habits of 2 populations of the province of Barcelona (I): design and validation of a semiquantitative questionnaire of frequency of food consumption. Med Clin 103, 1–4. 8051958

[23] Roddam AW , Spencer E , Banks E et al. (2005) Reproducibility of a short semi-quantitative food group questionnaire and its performance in estimating nutrient intake compared with a 7-day diet diary in the Million Women Study. Public Health Nutr 8, 201–213. 15877913 10.1079/phn2004676

[24] Babíc D , Sindik J & Missoni S (2014) Development and validation of a self-administered food frequency questionnaire to assess habitual dietary intake and quality of diet in healthy adults in the republic of Croatia. Coll Antopol 38, 1017–1026. 25420388

[25] Rothenberg E (1994) Validation of the food frequency questionnaire with the 4-day record method and analysis of 24-h urinary nitrogen. Eur J Clin Nutr 48, 725–735. 7835327

[26] Schröder H , Covas MI , Marrugat J et al. (2001) Use of a three-day estimated food record, a 72-hour recall and a food-frequency questionnaire for dietary assessment in a Mediterranean Spanish population. Clin Nutr 20, 429–37. 11534938 10.1054/clnu.2001.0460

[27] Tjønneland A , Overvad K , Haraldsdóttir J et al. (1991) Validation of a semiquantitative food frequency questionnaire developed in Denmark. Int J Epidemiol 20, 906–912. 1800429 10.1093/ije/20.4.906

[28] Van Dongen M , Lentjes M , Wijckmans N et al. (2011) Validation of a food-frequency questionnaire for Flemish and Italian-native subjects in Belgium: the IMMIDIET study. Nutrition 27, 302–309. 20579850 10.1016/j.nut.2010.02.006

[29] Gazan R , Vieux F , Darmon N et al. (2017) Structural validation of a French food frequency questionnaire of 94 items. Front Nutr 4, 62. 10.3389/fnut.2017.00062 PMC5742348 29326941

[30] Bonifacj C , Gerber M , Scali J et al. (1997) Comparison of dietary assessment methods in a southern French population: use of weighed records, estimated-diet records and a food-frequency questionnaire. Eur J Clin Nutr 51, 217–231. 9104572 10.1038/sj.ejcn.1600387

[31] Dumartheray EW , Krieg MA , Cornuz J et al. (2006) Validation and reproducibility of a semi-quantitative food frequency questionnaire for use in elderly Swiss women. J Hum Nutr Diet 19, 321–330. 16961678 10.1111/j.1365-277X.2006.00721.x

[32] Fernández-Ballart JD , Piñol JL , Zazpe I et al. (2010) Relative validity of a semi-quantitative food-frequency questionnaire in an elderly Mediterranean population of Spain. Br J Nutr 103, 1808–1816. 20102675 10.1017/S0007114509993837

[33] Fidanza F , Gentile MG & Porrini M (1995) A self-administered semiquantitative food-frequency questionnaire with optical reading and its concurrent validation. Eur J Epidemiol 11, 163–170. 7672070 10.1007/BF01719482

[34] Fregapane G & Asensio-García C (2000) Dietary assessment of an educated young Spanish population using a self-administered meal-based food frequency questionnaire. Eur J Epidemiol 16, 183–191. 10845270 10.1023/a:1007630521750

[35] Friis S , Kjaer S , Stripp C et al. (1997) Reproducibility and relative validity of a self-administered semiquantitative food frequency questionnaire applied to younger women. J Clin Epidemiol 50, 303–311. 9120530 10.1016/s0895-4356(96)00379-4

[36] Grootenhuis P , Westenbrink S , Sie CMTL et al. (1995) A semiquantitative food frequency questionnaire for use in epidemiologic research among the elderly: validation by comparison with dietary history. J Clin Epidemiol 48, 859–868. 7782793 10.1016/0895-4356(95)00013-t

[37] Dehghan M , del Cerro S , Zhang X et al. (2012) Validation of a semi-quantitative food frequency questionnaire for Argentinean adults. PLoS One 7, 1–9. 10.1371/journal.pone.0037958 PMC3360668 22662256

[38] Nath SD & Huffman FG (2005) Validation of a semiquantitative food frequency questionnaire to assess energy and macronutrient intakes of Cuban Americans. Int J Food Sci Nutr 56, 309–314. 16236592 10.1080/09637480500284993

[39] Rimm EB , Giovannucci EL , Stampfer MJ et al. (1992) Reproducibility and validity of an expanded self-administered semiquantitative food frequency questionnaire among male health professionals. Am J Epidemiol 135, 1114–1126. 1632423 10.1093/oxfordjournals.aje.a116211

[40] Rodríguez MM , Méndez H , Torún B et al. (2002) Validation of a semi-quantitative food-frequency questionnaire for use among adults in Guatemala. Public Health Nutr 5, 691–699. 12372164 10.1079/PHN2002333

[41] Shatenstein B , Nadon S , Godin C et al. (2005) Development and validation of a food frequency questionnaire. Can J Diet Pr Res 66, 67–75. 10.3148/66.2.2005.67 15975195

[42] Willet WC , Sampson L , Stampfer MJ et al. (1985) Reproducibility and validity of a semiquantitative food frequency questionnaire. Am J Epidemiol 122, 51–65. 4014201 10.1093/oxfordjournals.aje.a114086

[43] Yuan C , Spiegelman D , Rimm EB et al. (2017) Validity of a dietary questionnaire assessed by comparison with multiple weighed dietary records or 24-hour recalls. Am J Epidemiol 185, 570–584. 28338828 10.1093/aje/kww104 PMC5859994

[44] Sanjeevi N , Freeland-Graves J & George GC (2017) Relative validity and reliability of a 1-week, semiquantitative food frequency questionnaire for women participating in the supplemental nutrition assistance program. J Acad Nutr Diet 117, 1972–1982.e2. Elsevier B.V. 28669810 10.1016/j.jand.2017.05.013

[45] Denova-Gutiérrez E , Ramírez-Silva I , Rodríguez-Ramírez S et al. (2016) Validity of a food frequency questionnaire to assess food intake in Mexican adolescent and adult population. Salud Publica Mex 58, 617–628. 28225938 10.21149/spm.v58i6.7862

[46] Feskanich D , Marshall J , Rimm EB et al. (1994) Simulated validation of a brief food frequency questionnaire. Ann Epidemiol 4, 181–187. 8055118 10.1016/1047-2797(94)90095-7

[47] Hernández-Avila M , Romieu I , Parra S et al. (1998) Validity and reproducibility of a food frequency questionnaire to assess dietary intake of women living in Mexico City. Salud Publica Mex 40, 133–140. 9617194 10.1590/s0036-36341998000200005

[48] Jackson M , Walker S , Cade J et al. (2001) Reproducibility and validity of a quantitative food-frequency questionnaire among Jamaicans of African origin. Public Health Nutr 4, 971–980. 11784410 10.1079/phn2001166

[49] Kumanyika SK , Tell GS , Shemanski L et al. (1997) Dietary assessment using a picture-sort approach. Am J Clin Nutr 65, S1123–S1129. 10.1093/ajcn/65.4.1123S 9094908

[50] Lee MM , Lee F & Wang S (1994) A semiquantitative dietary history questionnaire for Chinese Americans. Ann Epidemiol 4, 188–197. 8055119 10.1016/1047-2797(94)90096-5

[51] Longnecker MP , Lissner L , Holden JM et al. (1993) The reproducibility and validity of a self-administered semiquantitative food frequency questionnaire in subjects from South Dakota and Wyoming. Epidemiology 4, 356–365. 8347747 10.1097/00001648-199307000-00012

[52] Macedo-Ojeda G , Vizmanos-Lamotte B , Márquez-Sandoval YF et al. (2013) Validation of a semi-quantitative food frequency questionnaire to assess food groups and nutrient intake. Nutr Hosp 28, 2212–2220. 24506403

[53] Bowen L , Bharathi AV , Kinra S et al. (2012) Short communication development and evaluation of a semi-quantitative food frequency questionnaire for use in urban and rural India. Asia Pac J Clin Nutr 21, 355–360. 22705424

[54] Chen Y , Ahsan H , Parvez F et al. (2004) Validity of a food-frequency questionnaire for a large prospective cohort study in Bangladesh. Br J Nutr 92, 851–859. 15533275 10.1079/bjn20041277

[55] Bijani A , Esmaili H , Ghadimi R et al. (2018) Development and validation of a semi-quantitative food frequency questionnaire among older people in north of Iran. Casp J Intern Med 9, 78–86. 10.22088/cjim.9.1.78 PMC5771365 29387324

[56] Jayawardena R , Byrne NM , Soares MJ et al. (2016) Validity of a food frequency questionnaire to assess nutritional intake among Sri Lankan adults. Springerplus 5, 162. 27026859 10.1186/s40064-016-1837-x PMC4766149

[57] Ke L , Toshiro T , Fengyan S et al. (2005) Relative validity of a semi-quantitative food frequency questionnaire versus 3 day weighed diet records in middle-aged in habitants in Chaoshan Area, China. Asian Pac J Cancer Prev 6, 376–381. 16236003

[58] Park MK , Noh HY , Song NY et al. (2012) Validity and reliability of a dish-based, semi-quantitative food frequency questionnaire for Korean diet and cancer. Asian Pac J Cancer Prev 13, 545–552. 22524822 10.7314/apjcp.2012.13.2.545

[59] Sudha V , Radhika G , Sathya RM et al. (2006) Reproducibility and validity of an interviewer-administered semi-quantitative food frequency questionnaire to assess dietary intake of urban adults in southern India. Int J Food Sci Nutr 57, 481–493. 17162327 10.1080/09637480600969220

[60] Tokudome S , Imaeda N , Tokudome Y et al. (2001) Original communication relative validity of a semi-quantitative food frequency questionnaire versus 28 day weighed diet records in Japanese female dietitians. Eur J Clin Nutr 55, 735–742. 11528486 10.1038/sj.ejcn.1601215

[61] Yang YJ , Kim MK , Hwang SH et al. (2010) Relative validities of 3-day food records and the food frequency questionnaire. Nutr Resp Pr 4, 142–148. 10.4162/nrp.2010.4.2.142 PMC2867225 20461203

[62] Whitton C , Ho JCY , Tay Z et al. (2017) Relative validity and reproducibility of a food frequency questionnaire for assessing dietary intakes in a multi-ethnic Asian population using 24-h dietary recalls and biomarkers. Nutrients 9, 1059. 10.3390/nu9101059 PMC5691676 28946670

[63] Aoun C , Daher RB , Osta N et al. (2019) Reproducibility and relative validity of a food frequency questionnaire to assess dietary intake of adults living in a Mediterranean country. PLoS One 14, e0218541. 31206566 10.1371/journal.pone.0218541 PMC6576765

[64] Barrett JS & Gibson PR (2010) Development and validation of a comprehensive semi-quantitative food frequency questionnaire that includes FODMAP intake and glycemic index. J Am Diet Assoc 110, 1469–1476. 20869485 10.1016/j.jada.2010.07.011

[65] Fayet F , Flood V , Petocz P et al. (2011) Relative and biomarker-based validity of a food frequency questionnaire that measures the intakes of vitamin B 12, folate, iron, and zinc in young women. Nutr Res 31, 14–20. 21310301 10.1016/j.nutres.2010.12.004

[66] Horwath C (1993) Validity of a short food frequency questionnaire for estimating nutrient intake in elderly people. Br J Nutr 70, 3–14. 8399110 10.1079/bjn19930100

[67] Smith W , Mitchell P , Reay EM et al. (1998) Validity and reproducibility of a self-administered food frequency questionnaire in older people. Aust N Z J Public Health 22, 456–463. 9659773 10.1111/j.1467-842x.1998.tb01414.x

[68] Beck KL , Houston ZL , McNaughton SA et al. (2020) Development and evaluation of a food frequency questionnaire to assess nutrient intakes of adult women in New Zealand. Nutr Diet 77, 253–259. 30277640 10.1111/1747-0080.12472 PMC7187395

[69] Zack RM , Irema K , Kazonda P et al. (2018) Validity of an FFQ to measure nutrient and food intakes in Tanzania. New Testam Stud 21, 2211–2220. 10.1017/S1368980018000848 PMC6101256 29656731

[70] Gunes FE , Imeryuz N , Akalin A et al. (2015) Development and validation of a semi-quantitative food frequency questionnaire to assess dietary intake in Turkish adults. J Pak Med Assoc 65, 756–763. 26160087

[71] Martínez RM , Tuya LC , Martínez M et al. (2009) El coeficiente de correlacion de los rangos de Spearman caracterización [The correlation coefficient of the Spearman ranges. Characterization]. Rev Habanera Ciencias Médicas 7, 1–19.

[72] Cortés-Reyes E , Rubio-Romero JA & Gaitán-Duarte H (2010) Statistical methods for evaluating diagnostic test agreement and reproducibility. Rev Colomb Obstet Ginecol 61, 247–255.

[73] Pértega Díaz S & Pita Fernández S (2002) Determinación del tamaño muestral para calcular la significación del coeficiente de correlación lineal [Determination of the sample size to calculate the significance of the linear correlation coefficient]. Cad Atención Primaria 9, 209–211.

[74] Martínez-González M , Toledo E & Sánchez-Villegas A (2014) Análisis de concordancia, validez y pronóstico [Analysis of concordance, validity and prognosis]. In Bioestadistica Amigable Friendly Bioestatistics, 3rd ed., pp. 455–486 [ M Martínez-González , A Sánchez-Villegas , E Toledo-Atucha , et al., editors]. Barcelona: Elsevier.

[75] Pita S & Pértegas S (2003) La fiabilidad de las mediciones clínicas: el análisis de concordancia para variables numéricas [The reliability of clinical measurements: the concordance analysis for numerical variables]. Fisterra 10, 290–296.

[76] Cade J , Thompson R , Burley V et al. (2002) Development, validation and utilisation of food-frequency questionnaires – a review. Public Health Nutr 5, 567–587. 12186666 10.1079/PHN2001318

[77] Sánchez-Villegas A , Martín-Calvo N & Martínez-González M (2014) Correlación y regresión lineal simple [Correlation and simple linear regression]. In Bioestadistica Amigable Friendly Bioestatistics, 3rd ed., pp. 269–326 [ M Martínez-González , A Sánchez-Villegas , E Toledo-Atucha et al., editors]. Barcelona: Elsevier.

[78] Wakai K (2009) A review of food frequency questionnaires developed and validated in Japan. J Epidemiol 19, 1–11. 19164867 10.2188/jea.JE20081007 PMC3924089

[79] Cade JE , Burley VJ , Warm DL et al. (2004) Food-frequency questionnaires: a review of their design, validation and utilisation. Nutr Res Rev 17, 5–22. 19079912 10.1079/NRR200370

[80] Serra-Majem L , Frost Andersen L , Henríque-Sánchez P et al. (2009) Evaluating the quality of dietary intake validation studies. Br J Nutr 102, S3–S9. 20100366 10.1017/S0007114509993114

[81] Dennis LK , Snetselaar LG , Nothwehr FK et al. (2003) Developing a scoring method for evaluating dietary methodology in reviews of epidemiologic studies. J Am Diet Assoc 103, 483–487. 12669012 10.1053/jada.2003.50081

