# Supplementary material for: Validation of semiquantitative FFQ administered to adults: a systematic review
Source: Public Health Nutr. 2020 Aug 10;24(11):3399–418. doi: 10.1017/S1368980020001834 (PMC8314921; doi:10.1017/S1368980020001834)
Supplement: Supplementary file 1 [file S1368980020001834sup.zip › S1368980020001834sup001.docx]

**Supplemental Table 2. Nutrient validity results that were analyzed most frequently in the included studies, ordered chronologically.**

| **Reference** | **Correlation coefficients** | **Cholesterol** | | **SFA** | | **PUFA** | | **Fiber** | | **Vitamin C** | | **Calcium** | | **Iron** | |
| --- | --- | --- | --- | --- | --- | --- | --- | --- | --- | --- | --- | --- | --- | --- | --- |
|  |  | **UA** | **A^a^** | **UA** | **A^a^** | **UA** | **A^a^** | **UA** | **A^a^** | **UA** | **A^a^** | **UA** | **A^a^** | **UA** | **A^a^** |
| Willet et al. (1985)^(42)^ | Pearson^b^ | 0.52 | 0.61 | 0.44 | 0.59 | 0.4 | 0.48 | 0.46 | 0.58 | 0.63 | 0.66 | ND | ND | ND | ND |
| Willet et al. (1987)^(13)^ | Pearson^b^ | 0.67 | 0.38;  0.59^c^; 0.43^d^ | 0.74 | 0.60; 0.58^c^;  0.62^d^ | ND | ND | 0.44 | 0.61; 0.37^c^; 0.65^d^ | 0.38 | 0.46; 0.34^c^; 0.49^d^ | 0.63 | 0.55; 0.42^c^; 0.57^d^ | 0.47 | 0.38;  0.28^c^; 0.40^d^ |
| Tjønneland et al. (1991)^(27)^ | Pearson | M: 0.41; W: 0.05 | M: 0.50;  W: 0.04 | M: 0.42; W: 0.26 | M: 0.46;  W: 0.39 | M: 0.53; W: 0.28 | M: 0.60; W: 0.31 | M: 0.29; W: 0.38 | M: 0.39;  W: 0.53 | M: 0.64; W: 0.45 | M: 0.64;  W: 0.51 | M: 0.56; W: 0.20 | M: 0.71;  W: 0.39 | M: 0.44;  W: 0.38 | M: 0.56; W: 0.48 |
| Rimm et al. (1992)^(39)^ | Pearson^b^ | 0.62 | 0.67; D: 0.76 | 0.63 | 0.71; D: 0.75 | 0.33 | 0.29; D: 0.37 | 0.49 | 0.64; D: 0.68 | 0.64 | 0.68; D: 0.77 | 0.52 | 0.53; D: 0.60 | 0.28 | 0.28; D: 0.32 |
| Horwath (1993)^(66)^ | Pearson | M: 0.55; W: 0.64 | ND | M: 0.74; W: 0.66 | ND | M: 0.52; W: 0.57 | ND | M: 0.67; W: 0.59 | ND | M: 0.58; W: 0.39 | ND | M: 0.75; W: 0.62 | ND | ND | ND |
| Longnecker et al. (1993)^(51)^ | Pearson^b^ | 0.37; 0.41^c^ | 0.42; 0.63^c^ | 0.36; 0.40^c^ | 0.38; 0.56^c^ | 0.37; 0.29^c^ | 0.28; 0.43^c^ | 0.44; 0.37^c^ | 0.44; 0.49^c^ | 0.5; 0.43^c^ | 0.5; 0.46^c^ | 0.6; 0.57^c^ | 0.57; 0.69^c^ | 0.35; 0.43^c^ | 0.42; 0.52^c^ |
| Martín et al. (1993)^(5)^ | Pearson^b^ | 0.55 | 0.53; D: 0.62 | 0.47 | 0.40; D: 0.53 | 0.45 | 0.37; D: 0.49 | 0.51 | 0.51; D: 0.54 | 0.63 | 0.56; D: 0.71 | ND | ND | ND | ND |
| Feskanich et al. (1994)^(46)^ | Pearson^b^ | 0.34 | 0.39; D: 0.46 | 0.5 | 0.53; D: 0.60 | 0.16 | 0.18; D: 0.22 | 0.32 | 0.58; D: 0.65 | 0.53 | 0.65; D: 0.76 | 0.39 | 0.5; D: 0.60 | 0.08 | 0.1; D: 0.12 |
| Lee et al. (1994)^(50)^ | Pearson | 0.51 | ND | 0.37 | ND | ND | ND | 0.28 | ND | 0.5 | ND | 0.66 | ND | ND | ND |
| Porrini et al. (1994)^(21)^ | Spearman | 0.71 | ND | ND | ND | ND | ND | 0.45 | ND | 0.44; ^e^B: 0.12 | ND | ND | ND | 0.74 | ND |
| Ramón et al. (1994)^(22)^ | Pearson^b^ | 0.32; D: 0.44 | 0.35 | 0.41; D: 0.57 | 0.53 | 0.39; D: 0.49 | 0.41 | 0.23; D: 0.39 | 0.27 | 0.43; D: 0.38 | 0.48 | ND | ND | ND | ND |
| Rothenberg (1994)^(25)^ | Pearson | ND | ND | ND | ND | ND | ND | 0.48 | ND | 0.53 | ND | 0.5 | ND | 0.5 | ND |
| Fidanza et al. (1995)^(33)^ | Spearman | 0.59 | ND | ND | ND | ND | ND | 0.34 | ND | 0.33 | ND | 0.47 | ND | 0.59 | ND |
| Gnardellis et al. (1995)^(12)^ | Person^b,f,g^ | M: 0.63; W: 0.37 | D: M: 0.60; W: 0.28 | M: 0.52; W: 0.34 | D: M: 0.39; W: 0.64 | M: 0.39; W: 0.24 | D: M: 0.26; W: 0.37 | M: 0.18; W: 0.25 | D: M: 0.20; W: 0.19 | M: 0.30; W: 0.21 | D: M: 0.46; W: 0.17 | ND | ND | M: 0.31; W: 0.31; | D: M: 0.32 W: 0.29 |
| Grootenhuis et al. (1995)^(36)^ | Pearson | M: 0.63; W: 0.37 | D: M: 0.60; W: 0.28 | M: 0.52; W: 0.34 | D: M: 0.39; W: 0.64 | M: 0.39 W: 0.24 | D: M: 0.26; W: 0.37 | M: 0.18 W: 0.25 | D: M: 0.20; W: 0.19 | M: 0.30 W: 0.21 | D: M: 0.46; W: 0.17 | ND | ND | M: 0.31; W: 0.31 | D: M: 0.32; W: 0.29 |
| Bonifacj et al. (1997)^(30)^ | Pearson^b^ | 0.45 | 0.63; D: 0.52 | 0.27 | 0.42; D: 0.30 | 0.66 | 0.51; D: 0.70 | 0.36 | 0.54; D: 0.38 | 0.24 | 0.35; D: 0.28 | 0.29 | 0.58; D: 0.32 | 0.52 | 0.80; D: 0.59 |
| Friis et al. (1997)^(35)^ | Pearson^b,f^ | 0.38 | 0.45; D: 0.64 | ND | ND | ND | ND | 0.59 | 0.65; D: 0.73 | 0.53 | 0.55; D: 0.68 | 0.54 | 0.56; D: 0.64 | 0.27 | 0.40; D: 0.59 |
| Kumanyika et al. (1997)^(49)^ | Pearson | ND | D: 0.73^c^ | ND | D: 0.72^c^ | ND | ND | ND | D: 0.58 | ND | D: 0.24^c^ | ND | D: 0.24^c^ | ND | D: 0.24^c^ |
| Ocké et al. (1997)^(20)^ | Pearson^b^ | ND | ND | ND | ND | ND | ND | M: 0.51; D: 0.56. W: 0.67; D: 0.75 | M: 0.55; D: 0.61. W: 0.65; D: 0.74 | M: 0.39; D: 0.45. W: 0.58; D: 0.69 | M: 0.37; D: 0.43. W: 0.61; D: 0.71 | ND | ND | ND | ND |
| Hérnandez et al. (1998)^(47)^ | Pearson^b,f^ | 0.5; D: 0.56 | 0.48 | 0.5; D: 0.71 | 0.67 | 0.43; D: 0.21 | 0.18 | 0.56; D: 0.40 | 0.60 | 0.41; D: 0.49 | 0.44 | 0.61; D: 0.60 | 0.55 | 0.39; D: 0.36 | 0.26 |
| Klipstein et al. (1998)^(14)^ | ICC | 0.77 | ND | 0.64 | ND | 0.67 | ND | 0.67 | ND | 0.75 | ND | 0.79 | ND | 0.65 | ND |
|  | Pearson | 0.53 | 0.48^d^;  D: 0.59 | 0.49 | 0.39^d^; D: 0.52 | 0.57 | 0.52^d^; D: 0.62 | 0.64 | 0.59^d^; D: 0.62 | 0.68 | 0.64^d^; D: 0.70 | 0.73 | 0.70^d^; D: 0.72 | 0.67 | 0.42^d^; D: 0.44 |
| Smith et al. (1998)^(67)^ | Pearson | 0.51 | 0.56 | 0.47 | 0.66 | 0.48 | 0.54 | 0.38 | 0.53 | 0.68 | 0.70 | 0.55 | 0.61 | 0.25 | 0.41 |
|  | Spearman | ND | 0.60 | ND | 0.66 | ND | 0.51 | ND | 0.57 | ND | 0.69 | ND | 0.61 | ND | 0.37 |
| Fregapane et al. (2000)^(34)^ | Spearman | 0.55 | ND | 0.693 | ND | 0.305 | ND | 0.453 | ND | 0.312 | ND | 0.651 | ND | 0.475 | ND |
|  | ICC | 0.496 | ND | 0.584 | ND | 0.541 | ND | 0.575 | ND | 0.374 | ND | 0.68 | ND | 0.341 | ND |
| Jackson et al. (2001)^(48)^ | Pearson^b^ | ND | ND | 0.35 | 0.33 | 0.27 | 0.28 | ND | ND | 0.3 | 0.31 | 0.43 | 0.4 | 0.46 | 0.39 |
| Schröder et al. (2001)^(26)^ | Pearson | ND | ND | ND | ND | ND | ND | 0.33 | ND | 0.6 | ND | ND | ND | ND | ND |
|  | Spearman | ND | ND | ND | ND | ND | ND | 0.33 | ND | 0.48 | ND | ND | ND | ND | ND |
| Tokudome et al. (2001)^(60)^ | Pearson | 0.55 | ND | 0.62 | ND | 0.43 | ND | 0.62 | ND | 0.42 | ND | 0.52 | ND | 0.44 | ND |
|  | Pearson^b^ | 0.60 | 0.55; D: 0.59 | 0.62 | 0.58; D: 0.62 | 0.42 | 0.26; D: 0.28 | 0.61 | 0.64; D: 0.65 | 0.40 | 0.40; D: 0.42 | 0.53 | 0.62; D: 0.64 | 0.48 | 0.53; D: 0.55 |
|  | Spearman | 0.54 | 0.44 | 0.63 | 0.59 | 0.36 | 0.26 | 0.57 | 0.63 | 0.36 | 0.31 | 0.55 | 0.64 | 0.50 | 0.52 |
| Rodriguez et al. (2002)^(40)^ | Pearson | ND | ND | ND | ND | ND | ND | ND | ND | 0.12^b^; D: 0.22^b^ | 0.11^b^; D: 0.29^b^ | 0.52^b^; D: 0.67^b^ | 0.43^b^; D: 0.84^b^ | 0.38^b^; D: 0.45^b^ | 0.21^b^; D: 0.38^b^ |
| Masson et al. (2003)^(16)^ | Pearson^b^ | ND | M: 0.53; W: 0.51 | ND | M: 0.55; W: 0.81 | ND | M: 0.08; W: 0.68 | ND | ND | ND | M: 0.64; W: 0.68 | ND | M: 0.52; W: 0.78 | ND | M: 0.63; W: 0.64 |
|  | Spearman | ND | M: 0.55; W: 0.39 | ND | M: 0.59; W: 0.71 | ND | M: -0.07; W: 0.58 | ND | ND | ND | M: 0.48; W: 0.59 | ND | M: 0.49; W: 0.75 | ND | M: 0.60;  W: 0.54 |
| Moreira et al. (1998)^(17)^ | Pearson | M: 0.36; W: 0.21 | M: 0.32; W: 0.25^b^ | M: 0.39; W: 0.51 | M: 0.61; W: 0.53^b^ | M: 0.24; W: 0.47 | M: 0.25; W: 0.52^b^ | M: 0.52; W: 0.69 | M: 0.46; W: 0.71^b^ | M: 0.61; W: 0.45 | M: 0.52; W: 0.51^b^ | M: 0.61; W: 0.60 | M: 0.53; W: 0.61^b^ | M: 0.49; W: 0.33 | M: 0.43; W: 0.45^b^ |
| Chen et al. (2004)^(54)^ | Pearson^b^ | A: 0.14 | A: 0.14; D: 0.32 | 0.32 | A: 0.38; D: 0.76 | 0.16 | A: 0.26; D: 0.49 | A: 0.13; I: 0.19 | A: 0.17; D: 0.24. I: 0.18; D: 0.40 | A: 0.08; I: 0.06 | A: 0.05; D: 0.12 I: 0.02 | A: 0.19; I: 0.12 | A: 0.14; D: 0.23. I: 0.10; D: 0.17 | A: 0.14; I: 0.13 | A: 0.15; D: 0.28 I: 0.18; D: 0.34 |
| Ke et al. (2005)^(57)^ | Pearson | 0.41 | 0.51 | 0.51 | 0.52 | 0.38 | 0.44 | 0.19 | 0.18 | 0.23 | 0.37 | 0.34 | 0.49 | 0.29 | 0.36 |
|  | Pearson^b^ | 0.49 | 0.56 | 0.63 | 0.61 | 0.48 | 0.58 | 0.17 | 0.20 | 0.57 | 0.60 | 0.52 | 0.63 | 0.31 | 0.33 |
|  | Spearman | 0.46 | 0.56 | 0.68 | 0.65 | 0.39 | 0.59 | 0.17 | 0.19 | 0.33 | 0.44 | 0.36 | 0.57 | 0.29 | 0.30 |
| Nath & Huffman (2005)^(38)^ | Pearson | 0.2 | 0.12 | 0.67 | 0.35 | 0.71 | 0.48 | 0.18 | 0.14 | ND | ND | ND | ND | ND | ND |
| Roddam et al. (2005)^(82)^ | Pearson | 0.59 | 0.34 | 0.5 | 0.56 | 0.18 | 0.27 | 0.56 | 0.62 | 0.61 | 0.61 | ND | ND | ND | ND |
| Shatenstein et al. (2005)^(41)^ | Spearman | 0.52; M: 0.39; W: 0.57 | ND | 0.57; M: 0.43: W: 0.58 | ND | 0.45; M: 0.47; W: 0.42 | ND | 0.44; M: 0.56; W: 0.36 | ND | 0.37; M: 0.39; W: 0.37 | ND | 0.46; M: 0.39; W: 0.49 | ND | 0.47; M: 0.32; W: 0.40 | ND |
| Dumartheray et al. (2006)^(31)^ | Pearson | ND | ND | ND | ND | ND | ND | 0.469; 0.512^b^ | 0.589; 0.552^b^ | 0.552; 0.555^b^ | 0.504 0.441^b^ | 0.377; 0.354^b^ | 0.442; 0.423^b^ | 0.492; 0.479^b^ | 0.512; 0.496^b^ |
| Sudha et al. (2006)^(59)^ | Pearson^b^ | 0.23 | 0.53; D: 0.65 | 0.28 | 0.51; D: 057 | 0.24 | 0.51; D: 0.59 | 0.64 | 0.51; D: 0.57 | 0.54 | 0.29; D: 0.35 | 0.22 | 0.24; D: 0.27 | 0.62 | 0.35; D: 0.42 |
| Nöthlings et al. (2007)^(19)^ | Pearson | 0.24^h,i^; 0.24^h,j^ | 0.62^h,i^; 0.75^h,j^ | 0.35^h,i^; 0.34^h,j^ | 0.75^h,i^; 0.72^h,j^ | 0.16^h,i^; 0.24^h,j^ | 0.30^h,i^; 0.38^h,j^ | 0.37^h,i^; 0.41^h,j^ | 0.65^h,i^; 0.65^h,j^ | 0.30^h,i^; 0.27^h,j^ | 0.52^h,i^; 0.41^h,j^ | 0.42^h,i^; 0.43^h,j^ | 0.81^h,i^; 0.71^h,j^ | 0.23^h,i^; 0.25^h,j^ | 0.50^h,i^; 0.46^h,j^ |
| Mullie et al. (2009)^(18)^ | Pearson^b^ | ND | 0.24 | ND | 0.14 | ND | 0.33 | ND | 0.33 | ND | 0.35 | ND | 0.43 | ND | 0.02 |
| Barret & Gibson (2010)^(64)^ | Spearman | ND | ND | ND | 0.44 | ND | 0.328 | ND | 0.585 | ND | 0.628 | ND | 0.562 | ND | 0.665 |
| Fernández et al. (2010)^(32)^ | Pearson | 0.37 | 0.23 | 0.52 | 0.61 | 0.43 | 0.42 | 0.49 | 0.60 | 0.65 | 0.68 | ND | ND | ND | ND |
|  | ICC | 0.54 | 0.37 | 0.68 | 0.75 | 0.60 | 0.60 | 0.66 | 0.75 | 0.78 | 0.80 | ND | ND | ND | ND |
| Yang et al. (2010)^(61)^ | Pearson^k^ | ND | 0.33-0.37^l^; D: 0.43-0.45^l^. 0.29-0.41ª,^l^ | ND | ND | ND | ND | ND | 0.24-0.27^l^; D: 0.27-0.32^l^; 0.23-0.28ª,^l^ | ND | 0.28-0.32^l^; D: 0.34-0.38^l^ 0.18-0.23ª,^l^ | ND | 0.23-0.26^l^; D: 0.28-0.32^l^; 0.27-0.37ª,^l^ | ND | 0.29-0.33^l^; D: 0.34-0.38^l^; 0.21-0.29^a,l^ |
| van Dongen et al. (2011)^(28)^ | Pearson | M: 0.29; W: 0.59 | ND | M: 0.21; W: 0.34 | ND | M: 0.41; W: 0.04 | ND | M: 0.76; W: 0.67 | ND | ND | ND | ND | ND | ND | ND |
| Bowen et al. (2012)^(53)^ | Spearman^b^ | ND | ND | 0.57 | 0.52; D: 0.75 | ND | ND | 0.57 | 0.43; D: 0.72 | ND | ND | ND | ND | ND | ND |
| Dehghan et al. (2012)^(37)^ | Pearson^b^ | U: 0.30; R: 0.30 | D^g^: U: 0.50; R; 0.57 | U: 0.40; R: 0.40 | D^g^: U: 0.52; R: ND | U: 0.25; R: 0.11 | D^g^: U: 0.48; R: ND | U: 0.28; R: 0.46 | D^g^: U: 0.33; R: 0.65 | U: 0.31; R: 0.26 | D^g^: U: 0.41; R: 0.35 | U: 0.35; R: 0.46 | D^g^: U: 0.49; R: 0.68 | U: 0.36; R: 0.37 | D^g^: U: 0.62; R: 0.63 |
| Park et al. (2012)^(58)^ | Spearman | ND | ND | ND | ND | ND | ND | ND | ND | 0.30;  M: 0.32; W: 0.29 | ND | 0.42;  M: 0.41; W: 0.45 | ND | 0.20;  M: 0.18; W: 0.20 | ND |
| Macedo et al. (2013)^(52)^ | Pearson^b^ | 0.47 | 0.32 | 0.55 | 0.32 | 0.36 | 0.12 | 0.29 | 0.4 | 0.32 | 0.42 | 0.56 | 0.62 | 0.41 | 0.38 |
|  | ICC^b^ | D: 0.64 | D: 0.49 | D: 0.71 | D: 0.49 | D: 0.52 | D: 0.21 | D: 0.44 | D: 0.58 | D: 0.49 | D: 0.6 | D: 0.7 | D: 0.77 | D: 0.57 | D: 0.61 |
| Babić et al. (2014)^(24)^ | Pearson^b^ | 0.48 | 0.44^i^ | 0.41 | 0.39^i^ | 0.29 | 0.17^i^ | 0.25 | 0.24^i^ | 0.32 | 0.25^i^ | 0.1 | 0.11^i^ | 0.1 | 0.10^i^ |
| Gunes et al. (2015)^(70)^ | Pearson^b^ | 0.325; D: 340 | 0.249 ^j^ | ND | ND | 0.329; D: 0.368 | 0.192^j^ | 0.365; D: 0.311 | 0.441^j^ | 0.081; D: 0.138 | 0.017^j^ | 0.283; D: 0.462 | 0.221^j^ | 0.314  D: 0.460 | 0.305^j^ |
| Denova et al. (2016)^(45)^ | Pearson^b^ | 0.4 | 0.32; D: 0.50 | 0.49 | 0.52; D: 0.61 | 0.27 | 0.21; D: 0.36 | 0.35 | 0.35; D: 0.40 | 0.33 | 0.38; D: 0.43 | 0.42 | 0.40; D: 0.47 | 0.25 | 0.28;  D: 0.35 |
| Jayawardena et al. (2016)^(56)^ | Pearson | 0.23 | ND | ND | ND | 0.37 | ND | 0.32 | ND | 0.21 | ND | 0.33 | ND | 0.25 | ND |
| Knudsen et al. (2016)^(15)^ | Pearson | ND | ND | ND | 0.51; D: 0.61 | ND | 0.41; D: 0.49 | ND | 0.63; D: 0.70 | ND | 0.36; D: 0.44 | ND | 0.41; D: 0.45 | ND | 0.48; D: 0.58 |
| Gazan et al. (2017)^(29)^ | Spearman | ND | ND | 0.86 | 0.86 | 0.88 | 0.88 | 0.87 | 0.77 | 0.86 | 0.85 | 0.84 | 0.77 | 0.78 | 0.59 |
| Sanjeevi et al. (2017)^(44)^ | Pearson^m^ | 0.43, D: 0.48 | ND | 0.58, D: 0.63 | ND | 0.52, D: 0.57 | ND | 0.56, D: 0.62 | ND | 0.50, D: 0.55 | ND | 0.64, D: 0.68 | ND | 0.47, D: 0.50 | ND |
| Whitton et al. (2017)^(62)^ | Pearson^b^ | ND | ND | 0.27^n^ | 0.26^n^, D: 0.38 ^n^ | 0.16^n^ | 0.15^n^, D: 0.31^n^ | 0.51^o^ | 0.47^o^, D: 0.56^o^ | 0.31^p^ | 0.32^p^, D: 0.43^p^ | 0.34^p^ | 0.32^p^, D: 0.57^p^ | 0.43^p^ | 0.41^p^, D: 0.64^p^ |
| Yuan et al. (2017)^(43)^ | Spearman (WR) | 0.50 | 0.42; 0.42^q^; D: 0.65 | 0.44 | 0.61; 0.62^q^; D: 0.69 | 0.28 | 0.47; 0.45^q^; D: 0.57 | 0.46 | 0.62; 0.64^q^; D: 0.66 | 0.49 | 0.52; 0.53^q^; D: 0.61 | 0.56 | 0.63; 0.61^q^; D: 0.68 | 0.35 | 0.48; 0.48^q^; D: 0.56 |
|  | Spearman (24HR) | 0.39 | 0.42; 0.42^q^; D: 0.68 | 0.44 | 0.54; 0.57^q^; D: 0.71 | 0.24 | 0.41; 0.40^q^; D: 0.70 | 0.41 | 0.51; 0.52^q^; D: 0.65 | 0.47 | 0.46; 0.46^q^; D: 0.62 | 0.48 | 0.55; 0.54^q^; D: 0.68 | 0.3 | 0.37; 0.39^q^; D: 0.55 |
| Bijani et al. (2018)^(55)^ | Pearson | M: 0.25; W: 0.13 | ND | M: 0.44; W: 0.46 | ND | M: -0.10; W: -0.01 | ND | M: 0.25; W: 0.19 | ND | M: 0.26; W: 0.26 | ND | M: 0.25; W: 0.41 | ND | M: 0.15; W: 0.10 | ND |
| Zack et al. (2018)^(69)^ | Rosner | 0.26 | 0.17, D: 0.26 | 0.18 | 0.04, D: 0.06 | 0.15 | 0.17, D: 0.26 | ND | ND | 0.11 | 0.12, D: 0.19 | 0.10 | 0.15, D: 0.26 | 0.04 | 0.18, D: 0.27 |
| Aoun et al. (2019)^(63)^ | Spearman or Pearson^e^ | ND | ND | 0.969 | 0.934 | 0.969 | 0.958 | 0.970 | 0.950 | 0.987 | 0.982 | ND | ND | 0.967 | 0.967 |
| Beck et al. (2019)^(68)^ | Spearman | 0.48 | 0.48 | 0.59 | 0.61 | 0.43 | 0.53 | 0.24 | 0.43 | 0.49 | 0.56 | 0.49 | 0.65 | 0.11 | 0.33 |

SFA: saturated fatty acids; PFA: polyunsaturated fatty acids; UA: unadjusted; A: adjusted; ND: Not described; M: men; W: women; D: deattenuated; B: biochemical analyses; ICC: intraclass correlation coefficient; U: urban; R: rural; DR, diet record; 24HR, 24 hour-recall.

^a^Adjusted by energy; ^b^With logarithmic scale transformation; ^c^Adjusted by sex and age; ^d^Adjusted by energy-age-sex; ^e^Pearson’s correlation was used for variables with normal distributions and Spearman’s for non-parametrically distributed variables; ^f^Coefficients from the second SFFQ are reported; ^g^It is not clear whether the deattenuated coefficient was for raw or energy-adjusted values; ^h^Male and female subjects averaged; ^i^Fitted portion size; ^j^Predefined portion size; ^k^Displayed as a range because CC is shown for each season of the year; ^l^Adjusted by sex; ^m^With logarithmic scale transformation for some nutrients; ^n^as percentage of energy; ^o^grams per 1000 kcal; ^p^miligrams per 1000 kcal. ^q^Energy density method (divides the nutrient portion by total energy intake).

NOTE: Fayet et al. (2011) is not described in this table because they did not analyze any of these nutrients.
