# Supplementary material for: Validation of semiquantitative FFQ administered to adults: a systematic review
Source: Public Health Nutr. 2020 Aug 10;24(11):3399–418. doi: 10.1017/S1368980020001834 (PMC8314921; doi:10.1017/S1368980020001834)
Supplement: Supplementary file 1 [file S1368980020001834sup.zip › S1368980020001834sup002.docx]

**Supplemental Table 1-a. Quality and bias risk assessments according to year of publication.**

| **Reference** | **The Newcastle-Ottawa Scale** | | | | | | **Cochrane** | | **Strobe statement** | | | | | | |
| --- | --- | --- | --- | --- | --- | --- | --- | --- | --- | --- | --- | --- | --- | --- | --- |
|  | **Selection** | | **Compara-bility** | **Outcome** | | | **Selective outcome reporting (notification bias):** | | **Methods** | | | | | **Results** | |
|  | Representativeness of the exposed cohort: *a) truly representative of the average adults in the community; *b) somewhat representative of the average adults in the community; c) selected group of users (volunteers); d) No description of the derivation of the cohort | Ascertainment of exposure (recalls or food records): *a) secure record (diet records); *b) structured interview c) written self-report; d) no description | Comparability of cohorts on the basis of the design or analysis: *a) study controls by energy (at least); *b) study controls for any additional factor (such as sex and age) | Assessment of outcome (SFFQ): *a) interview; *b) record linkage; c) self-report; d) no description | 2) Was follow-up long enough for outcomes to occur; *a) yes (follow-up was enough to assess validity: 6-12 months); b) no. | Adequacy of follow-up of cohorts; *a) complete follow-up; *b) subjects lost at follow-up unllikely to induce bias (<30% or with description provided of the lost); c) follow-up rate <30% and no description of the lost or follow-up lost >30% with description of the lost; d) no statement | Description | Risk-of-bias judgement | Describe the setting, locations, and relevant dates, including periods of recruitment, exposure, follow-up, and data collection | Give the eligibility criteria, and the sources and methods of selection of participants | For each variable of interest, give sources of data and details of methods of assessment (measurement with SFFQ) | Explain how the study size was arrived at and, If applicable, describe analytical methods taking account of sampling strategy | Explain how quantitative variables were handled in the analyses. If applicable, describe which groupings were chosen and why | Give characteristics of study participants (eg demographic, clinical, social) and information on exposures and potential confounders | Report other analyses done—eg analyses of subgroups and interactions, and sensitivity analyses |
| Willet et al. (1985)^(42)^ | *b | *a | *a | c | *a | *b | Results are presented as described in the methods section. | Low risk | Yes | Incomplete | Yes | No | Yes | No | Yes: quintiles, ICC, log transformation |
| Willet et al. (1987)^(13)^ | c | *a | *a and *b | c | *a | *b | Results are presented as described in the methods section. | Low risk | Yes | Incomplete | Incomplete | No | Yes | No | Yes: log transformation |
| Tjønneland et al. (1991)^(27)^ | *b | *a | *a | c | b | *b | Results are presented as described in the methods section. | Low risk | Yes | Incomplete | Yes | No | Yes | No | Yes: quintiles, Spearman, log transformation |
| Rimm et al. (1992)^(39)^ | *b | *a | *a | c | *a | d | Results are presented as described in the methods section. | Low risk | Yes | Incomplete | Incomplete | No | Yes | No | Yes: log transformation, deattenuation, quintiles, ICC, regression coefficientes, coefficientes of variation. |
| Horwath et al. (1993)^(66)^ | c | *a | None | c | b | *b | Results are presented as described in the methods section. | Low risk | Incomplete | Incomplete | Incomplete | No | Yes | Yes | Yes: log transformation quintiles |
| Longnecker et al. (1993)^(51)^ | c | c | *b | c | *a | d | Results are presented as described in the methods section. | Low risk | Yes | Incomplete | Yes | No | Yes | Yes | Yes: quartiles |
| Martin et al. (1993)^(5)^ | *b | *a | *a | *b | *a | *b | Results are presented as described in the methods section. | Low risk | Incomplete | Incomplete | Incomplete | No | Yes | Yes | Yes: deattenuation, log transformation, ICC, quintiles |
| Feskanich et al. (1994)^(46)^ | *b | *a | *a | c | *a | d | Results are presented as described in the methods section. | Low risk | Yes | Incomplete | Incomplete | No | Yes | No | Yes: deattenuation, log transformation |
| Lee et al. (1994)^(50)^ | c | *b | None | *a | b | *b | Results are presented as described in the methods section. | Low risk | Yes | Incomplete | Yes | No | Yes | Yes | Yes: T-test, Spearman (not presented because they were not different from Pearson), quartiles. |
| Porrini et al. (1994)^(21)^ | c | *a | None | c | b | d | Statistical anayses are not described and results description is brief. | High risk | No | No | No | No | No | Yes | Yes: Wilcoxon, Spearman |
| Ramón et al. (1994)^(22)^ | *b | *a | *a | *a | *a | *b | Results are presented as described in the methods section. | Low risk | Incomplete | No | Incomplete | Yes | Yes | Yes | Yes: deattenuation quintiles, log transformation |
| Rothenberg (1994)^(25)^ | d | *a | None | *a | b | d | Results are presented as described in the methods section; however, Bland-Altman plots are not clearly shown (they are presented in tables). | Not clear | Incomplete | No | Incomplete | No | Yes | Yes | Yes: biomarkers, log transformation, Bland-Altman, tertiles |
| Fidanza et al. (1995)^(46)^ | c | *a | None^a^ | c | Follow-up not described | d | Adjustment by energy is presented in study results, although it was not mentioned in the methods section. | Low risk | Incomplete | Yes | Yes | No | Yes | Yes | Yes: tertiles |
| Gnardellis et al. (1995)^(12)^ | c | *b | *a | c | *a | *b | Results are presented as described in the methods section. | Low risk | Yes | Yes | Yes | No | Yes | Incomplete | Yes: log transformation ICC, deattenuation, quintiles |
| Grootenhuis et al. (1995)^(36)^ | *b | *b | None | c | b | *b | Bland-Altman plots are mentioned in the methods section but they are not shown in the results section. | High risk | Yes | Incomplete | Yes | No | Yes | Incomplete | Yes: tertiles |
| Bonifacj et al. (1997)^(30)^ | c | c | *a | c | *a | *b | Results are presented as described in the methods section. | Low risk | Yes | Yes | Yes | No | Yes | Yes | Yes: Spearman, deattenuation, quartiles, log transformation, paired t-test. |
| Friis et al. (1997)^(35)^ | c | *a | *a | c | *a | *b | Results are presented as described in the methods section. | Low risk | Yes | No | Yes | No | Yes | Yes | Yes: deattenuation, log transformation, Spearman, quintiles |
| Kumanyika et al. (1997)^(49)^ | c | *b | *b | *a | *a | *b | Methods and results are presented in the same section and there is not a statistical anlyses section. | Not clear | Yes | Yes | Incomplete | No | Yes | No | Yes: log transformation, deattenuation, Kruskall Wallis. |
| Ocké et al. (1997)^(20)^ | *b | *b | *a | c | *a | *b | Results are presented as described in the methods section. | Low risk | Yes | Yes | Yes | No | Yes | Yes | Yes: log transformation, deattenuation, linear regression model, t-tests. |
| Hérnandez et al. (1998)^(47)^ | *b | *b | *a | *a | *a | c | Results are presented as described in the methods section. | Low risk | Incomplete | Incomplete | Yes | No | Yes | No | Yes: ICC, log transformation deattenuation |
| Klipstein et al. (1998)^(14)^ | *b | *a | *a and *b | *a and c) | *a | *b | Results are presented as described in the methods section. | Low risk | Yes | Yes | Yes | No | Yes | Yes | Yes: quintiles, log transformation |
| Smith et al. (1998)^(67)^ | *b | *a | *a | *b | *a | c | Results are presented as described in the methods section. | Low risk | Yes | Incomplete | Yes | No | Yes | Incomplete | Yes: log transformation kappa, quintiles |
| Fregapane & Asensio (2000)^(34)^ | c | *a | None | *b | b | c | Results are presented as described in the methods section. | Low risk | Incomplete | No | Yes | Yes | Yes | Yes | Yes: ICC |
| Jackson et al. (2001)^(48)^ | *b | *b | *a | *a | *a | *b | Results are presented as described in the methods section. | Low risk | Incomplete | Incomplete | Yes | No | Yes | Yes | Yes: quartiles, ICC |
| Schröder et al. (2001)^(26)^ | c | d | None | *b | b | d | Results are presented as described in the methods section. | Low risk | No | No | Incomplete | No | Yes | Yes | Yes: biomarkers, ICC, quartiles |
| Tokudome et al. (2001)^(60)^ | c | *a | *a | c | *a | *b | Results are presented as described in the methods section. | Low risk | Yes | No | Incomplete | No | Yes | Yes | Yes: log transformation, Spearman, tertiles, kappa, deattenuation |
| Rodríguez et al. (2002)^(40)^ | c | *b | *a | *a | b | *b | Bland-Altman plots are mentioned in the methods section but they are not shown in the results section. | High risk | Incomplete | Yes | Yes | No | Yes | Incomplete | Yes: quartiles, kappa, deattenuation |
| Masson et al. (2003)^(16)^ | c | *a | *a | *b | b | c | Results are presented as described in the methods section. | Low risk | Incomplete | Incomplete | Yes | No | Yes | Yes | Yes: tertiles, kappa, Spearman, Wilcoxon, log transformation. |
| Moreira et al. (2003)^(17)^ | c | c | *a | *a | b | c | Results are presented as described in the methods section. | Low risk | Incomplete | No | Yes | No | Yes | No | Yes: quintiles, Bland-Altman, concordance |
| Chen et al. (2004)^(54)^ | *b | *b | *a | *a | *a | *b | Results are presented as described in the methods section. | Low risk | Yes | Incomplete | Yes | No | Yes | Yes | Yes: log transformation deattenuation, quintiles, ICC. |
| Ke et al. (2005)^(57)^ | d | c | *a | d | b | c | Results are presented as described in the methods section. | Low risk | Incomplete | No | Incomplete | No | Yes | Yes | Yes: Kappa, tertiles, Spearman |
| Nath & Huffman (2005)^(38)^ | c | *a | *a | d | b | d | Results are presented as described in the methods section. | Low risk | Incomplete | No | Incomplete | No | Yes | Incomplete | Yes: paired t-test |
| Roddam et al. (2005)^(23)^ | *a | c | *a | c | *a | *a | The study have different sample sizes for their different objectives (although they are clealry specified in each analysis). | Low risk | Yes | Incomplete | Yes | No | Yes | Yes | Yes: kappa |
| Shatenstein et al. (2005)^(41)^ | *b | *b | None | *b | b | c | Results are presented as described in the methods section. | Low risk | Yes | Yes | Yes | No | Yes | Yes | Yes: quartiles |
| Dumartheray et al. (2006)^(31)^ | c | *a | *a | *b | *a | *b | Results are presented as described in the methods section. | Low risk | Yes | Incomplete | Yes | No | Yes | Yes | Yes: Bland-Altman, log transformation, tertiles |
| Sudha et al. (2006)^(59)^ | *b | *b | *a | *a | *a | *b | Results are presented as described in the methods section. | Low risk | Incomplete | Incomplete | Yes | No | Yes | Yes | Yes: deattenuation,gráficos Bland-Altman, ICC, quartiles |
| Nöthlings et al. (2007)^(19)^ | *b | *b | *a | *b | *a | *b | Results are presented as described in the methods section. | Low risk | Incomplete | Incomplete | Incomplete | No | Yes | Yes | Yes: deattenuation |
| Mullie et al. (2009)^(18)^ | c | *a | *a | d | b | *b | Results are presented as described in the methods section. | Low risk | Yes | Yes | Yes | No | Yes | Yes | Yes: tertiles, paired t-test, tertiles, kappa |
| Barret et al. (2010)^(64)^ | c | c | *a | c | *a | *b | Results are presented as described in the methods section. | Low risk | Incomplete | Yes | Incomplete | No | Yes | Yes | Yes: tertiles y kappa, ICC, Bland-Altman, ICC |
| Fernández et al. (2010)^(32)^ | *b | c | *a | c | *a | *a | Results are presented as described in the methods section. | Low risk | Incomplete | Yes | Yes | No | Yes | Yes | Yes: ICC, quintiles, Bland-Altman, log transformation, deattenuation |
| Yang et al. (2010)^(61)^ | c | *a | *a and *b | *a | *a | c | Results are presented as described in the methods section. | Low risk | Yes | Yes | Yes | No | Yes | Yes | Yes: deattenuation, paired t-test, log transformation, deattenuation, quartiles |
| Fayet et al. (2011)^(65)^ | c | *b | *a | c | b | *b | Deattenuated correlation coefficients are not shown for all nutrients (incomplete data). | High risk | Incomplete | Yes | Yes | No | Yes | Yes | Yes: Bland-Altman, deattenuation, quartiles, log transformation |
| van Dongen et al. (2011)^(28)^ | *b | *b | None | *b | *a | d | Results are presented as described in the methods section. | Low risk | Yes | Yes | Yes | No | Yes | Yes | Yes: Bland-Altman, quartiles |
| Bowen et al. (2012)^(53)^ | c | *b | *a | *a | b | d | Final sample size is not clearly stated. | High risk | Incomplete | Incomplete | Yes | No | Yes | Yes | Yes: deattenuation, Bland-Altman, kappa |
| Dehghan et al. (2012)^(37)^ | *b | *b | *a and *b | *a | *a | *b | Results are presented as described in the methods section. | Low risk | Incomplete | Incomplete | Yes | No | Yes | Yes | Yes: log transformation, deattenuation, Bland Altman, quartiles, ICC. |
| Park et al (2012)^(58)^ | c | c | *a | *a and c) | *a | *b | Results are presented as described in the methods section. | Low risk | Yes | Yes | Yes | No | Yes | Yes | Yes: quintiles |
| Macedo et al. (2013)^(52)^ | c | *a | *a | *a | *a | d | Results are presented as described in the methods section^b^ | Low risk | Incomplete | Incomplete | Yes | No | Yes | Yes | Yes: deattenuation, Bland Altman, ICC, quintiles. |
| Bábic et al. (2014)^(24)^ | d | c | *b | c | b | c | Food group correlation coefficients are not shown in the results section; in the tables, it is not clear whether Spearman or Pearson correlation coefficientes where performed. | High risk | Incomplete | Yes | Yes | No | Yes | Yes | Yes: Spearman |
| Gunes et al. (2015)^(70)^ | *b | *b | *a | *a | *a | c | Results are presented as described in the methods section. | Low risk | Yes | Yes | Yes | No | Yes | Yes | Yes: deattenuation, quartiles, Bland-Altman, kappa |
| Denova et al. (2016)^(45)^ | *b | *b | *a | *a | b | *b | Results are presented as described in the methods section. | Low risk | Incomplete | Yes | Yes | No | Yes | Yes | Yes: Bland-Altman, deattenuation, kappa, quartiles, log transformation, regression |
| Jayawarena et al. (2016)^(56)^ | *b | c | None | *a | b | *b | Results are presented as described in the methods section. | Low risk | Incomplete | Incomplete | Yes | No | Yes | Yes | Yes: Bland-Altman |
| Knudsen et al. (2016)^(15)^ | c | c | *a | c | b | *b | Results are presented as described in the methods section. | Low risk | Yes | Incomplete | Incomplete | No | Yes | Yes | Yes: Bland-Altman, deattenuation, quintiles, paired t-test, Wilcoxon |
| Gazan et al. (2017) ^(29)^ | d | d | *a | d | b | *b | Results are presented as described in the methods section | Low risk | No | No | No | No | Yes | No | Yes: kappa, quartiles |
| Sanjeevi et al. (2017) ^(44)^ | c | *a | None | *a | b | *a | It is not clear which correlation coefficients are log transformed (analysis performed with non-parametric data) and whic are not. | Not clear | Yes | Yes | Incomplete | Yes | Yes | Yes | Deattenuation, log-transformation (for non-parametric variables), paired t-test, Weighted Cohen’s kappa, quartile classification. |
| Whitton et al. (2017) ^(62)^ | *b | *b | *b | *a | b | *b | Results are presented as described in the methods section. | Low risk | No | Yes | Yes | Yes | Yes | Yes | Log transformation, deattenuation, ICC, ANOVA, chi-square. |
| Yuan et al. (2017)^(43)^ | *b | c | *a | c | *a | *b | Results are presented as described in the methods section. | Low risk | Yes | Yes | Yes | No | Yes | Yes | Yes: log transformation, Bland-Altman, Spearman |
| Bijani et al. (2018)^(55)^ | *b | *b | none | *a | b | *b | Results are presented as described in the methods section | Low risk | Incomplete | No | Yes | No | Yes | Yes | Yes: Pitman's test, Bland-Altman, Spearman |
| Zack et al. (2018)^(69)^ | *b | *b | *a | *a | b | *b | Results are presented as described in the methods section. | Low risk | Yes | Yes | Yes | No | Yes | Yes | Deattenuated energy-adjusted rank correlations for nutrients, deattenuated rank correlations for food groups, cross-classification analysis of energy-adjusted nutrient quartiles using percent agreement, kappa, Bland-Altman analysis, ICC. |
| Aoun et al. (2019)^(63)^ | *b | *b | *a | *a | b | *a | In the validation table the correlation coefficients Sp and P were used depending on their distribution but it's not specific. | Not clear | Yes | Yes | Yes | Yes | Yes | Incomplete | Yes: Bland-Altman, kappa, quartiles, ICC Sp |
| Beck et al. (2019)^(68)^ | *b | c | *a | c | b | *b | Results are presented as described in the methods section | Low risk | Yes | Incomplete | Yes | No | Yes | Yes | Yes: Bland-Altman, kappa, quartiles |

^a^ Authors mention in the results section (not in the methods section) that they performed energy adjustment correlation coefficients but that they do not show them because they are similar to raw data.

^b^ There is an error in energy-adjustment data acommodation in table II.

**Supplemental Table 1-b. Quality assessment and risk of bias of the included articles, ordered in accordance with those that had the highest number of met criteria elements and in chronological order.**

| **Reference** | **1** | **2** | **3** | **4** | **5** | **6** | **7** | **8** | **9** | **10** | **11** | **12** | **13** | **14** |
| --- | --- | --- | --- | --- | --- | --- | --- | --- | --- | --- | --- | --- | --- | --- |
| Klipstein et al. (1998)^(14)^ |  |  |  |  |  |  |  |  |  |  |  |  |  |  |
| Chen et al. (2004)^(54)^ |  |  |  |  |  |  |  |  |  |  |  |  |  |  |
| Ocké et al. (1997)^(20)^ |  |  |  |  |  |  |  |  |  |  |  |  |  |  |
| Gunes et al. (2015)^(70)^ |  |  |  |  |  |  |  |  |  |  |  |  |  |  |
| Whitton et al. (2017)^(62)^ |  |  |  |  |  |  |  |  |  |  |  |  |  |  |
| Ramón et al. (1994)^(22)^ |  |  |  |  |  |  |  |  |  |  |  |  |  |  |
| Jackson et al. (2001)^(48)^ |  |  |  |  |  |  |  |  |  |  |  |  |  |  |
| Sudha et al. (2006)^(59)^ |  |  |  |  |  |  |  |  |  |  |  |  |  |  |
| Dehghan et al. (2012)^(37)^ |  |  |  |  |  |  |  |  |  |  |  |  |  |  |
| Dumartheray et al. (2006)^(31)^ |  |  |  |  |  |  |  |  |  |  |  |  |  |  |
| Denova et al. (2016)^(45)^ |  |  |  |  |  |  |  |  |  |  |  |  |  |  |
| Aoun et al. (2019)^(63)^ |  |  |  |  |  |  |  |  |  |  |  |  |  |  |
| Yang et al. (2010)^(61)^ |  |  |  |  |  |  |  |  |  |  |  |  |  |  |
| van Dongen et al. (2011)^(28)^ |  |  |  |  |  |  |  |  |  |  |  |  |  |  |
| Yuan et al. (2017)^(43)^ |  |  |  |  |  |  |  |  |  |  |  |  |  |  |
| Zack et al. (2018)^(69)^ |  |  |  |  |  |  |  |  |  |  |  |  |  |  |
| Martin et al. (1993)^(5)^ |  |  |  |  |  |  |  |  |  |  |  |  |  |  |
| Nöthlings et al. (2007)^(19)^ |  |  |  |  |  |  |  |  |  |  |  |  |  |  |
| Smith et al. (1998)^(67)^ |  |  |  |  |  |  |  |  |  |  |  |  |  |  |
| Willet et al. (1985)^(42)^ |  |  |  |  |  |  |  |  |  |  |  |  |  |  |
| Gnardellis et al. (1995)^(12)^ |  |  |  |  |  |  |  |  |  |  |  |  |  |  |
| Roddam et al. (2005)^(23)^ |  |  |  |  |  |  |  |  |  |  |  |  |  |  |
| Fernández et al. (2010)^(32)^ |  |  |  |  |  |  |  |  |  |  |  |  |  |  |
| Park et al (2012)^(58)^ |  |  |  |  |  |  |  |  |  |  |  |  |  |  |
| Bonifacj et al. (1997)^(30)^ |  |  |  |  |  |  |  |  |  |  |  |  |  |  |
| Friis et al. (1997)^(35)^ |  |  |  |  |  |  |  |  |  |  |  |  |  |  |
| Shatenstein et al. (2005)^(41)^ |  |  |  |  |  |  |  |  |  |  |  |  |  |  |
| Mullie et al. (2009)^(18)^ |  |  |  |  |  |  |  |  |  |  |  |  |  |  |
| Kumanyika et al. (1997)^(49)^ |  |  |  |  |  |  |  |  |  |  |  |  |  |  |
| Hérnandez et al. (1998)^(47)^ |  |  |  |  |  |  |  |  |  |  |  |  |  |  |
| Macedo et al. (2013)^(52)^ |  |  |  |  |  |  |  |  |  |  |  |  |  |  |
| Sanjeevi et al. (2017)^(44)^ |  |  |  |  |  |  |  |  |  |  |  |  |  |  |
| Tjønneland et al. (1991)^(27)^ |  |  |  |  |  |  |  |  |  |  |  |  |  |  |
| Lee et al. (1994)^(50)^ |  |  |  |  |  |  |  |  |  |  |  |  |  |  |
| Tokudome et al. (2001)^(60)^ |  |  |  |  |  |  |  |  |  |  |  |  |  |  |
| Bijani et al. (2018)^(55)^ |  |  |  |  |  |  |  |  |  |  |  |  |  |  |
| Beck et al. (2019)^(68)^ |  |  |  |  |  |  |  |  |  |  |  |  |  |  |
| Willet et al. (1987)^(13)^ |  |  |  |  |  |  |  |  |  |  |  |  |  |  |
| Rimm et al. (1992)^(10)^ |  |  |  |  |  |  |  |  |  |  |  |  |  |  |
| Feskanich et al. (1994)^(46)^ |  |  |  |  |  |  |  |  |  |  |  |  |  |  |
| Rodríguez et al. (2002)^(40)^ |  |  |  |  |  |  |  |  |  |  |  |  |  |  |
| Masson et al. (2003)^(16)^ |  |  |  |  |  |  |  |  |  |  |  |  |  |  |
| Barret et al. (2010)^(64)^ |  |  |  |  |  |  |  |  |  |  |  |  |  |  |
| Jayawarena et al. (2016)^(56)^ |  |  |  |  |  |  |  |  |  |  |  |  |  |  |
| Longnecker et al. (1993)^(51)^ |  |  |  |  |  |  |  |  |  |  |  |  |  |  |
| Fregapane & Asensio (2000)^(34)^ |  |  |  |  |  |  |  |  |  |  |  |  |  |  |
| Fayet et al. (2011)^(65)^ |  |  |  |  |  |  |  |  |  |  |  |  |  |  |
| Grootenhuis et al. (1995)^(36)^ |  |  |  |  |  |  |  |  |  |  |  |  |  |  |
| Bowen et al. (2012)^(53)^ |  |  |  |  |  |  |  |  |  |  |  |  |  |  |
| Knudsen et al. (2016)^(15)^ |  |  |  |  |  |  |  |  |  |  |  |  |  |  |
| Fidanza et al. (1995)^(33)^ |  |  |  |  |  |  |  |  |  |  |  |  |  |  |
| Horwath et al. (1993)^(66)^ |  |  |  |  |  |  |  |  |  |  |  |  |  |  |
| Moreira et al. (2003)^(17)^ |  |  |  |  |  |  |  |  |  |  |  |  |  |  |
| Bábic et al. (2014)^(24)^ |  |  |  |  |  |  |  |  |  |  |  |  |  |  |
| Nath & Huffman (2005)^(38)^ |  |  |  |  |  |  |  |  |  |  |  |  |  |  |
| Rothenberg (1994)^(25)^ |  |  |  |  |  |  |  |  |  |  |  |  |  |  |
| Ke et al. (2005)^(57)^ |  |  |  |  |  |  |  |  |  |  |  |  |  |  |
| Schröder et al. (2001)^(26)^ |  |  |  |  |  |  |  |  |  |  |  |  |  |  |
| Gazan et al. (2017)^(29)^ |  |  |  |  |  |  |  |  |  |  |  |  |  |  |
| Porrini et al. (1994)^(21)^ |  |  |  |  |  |  |  |  |  |  |  |  |  |  |
